# Testing the applicability of a benthic foraminiferal-based transfer function for the reconstruction of paleowater depth changes in Rhodes (Greece) during the early Pleistocene

**DOI:** 10.1371/journal.pone.0188447

**Published:** 2017-11-22

**Authors:** Yvonne Milker, Manuel F. G. Weinkauf, Jürgen Titschack, Andre Freiwald, Stefan Krüger, Frans J. Jorissen, Gerhard Schmiedl

**Affiliations:** 1 Center for Earth System Research and Sustainability, Institute for Geology, University of Hamburg, Hamburg, Germany; 2 Department of Earth Sciences, University of Geneva, Genève, Switzerland; 3 MARUM—Center for Marine Environmental Sciences, University Bremen, Bremen, Germany; 4 Department for Marine Research, Senckenberg am Meer, Wilhelmshaven, Germany; 5 Institute for Geophysics and Geology, University of Leipzig, Leipzig, Germany; 6 UMR CNRS 6112 LPG-BIAF Bio-Indicateurs Actuels et Fossiles, Université d'Angers, France; Universita degli Studi di Urbino Carlo Bo, ITALY

## Abstract

We present paleo-water depth reconstructions for the Pefka E section deposited on the island of Rhodes (Greece) during the early Pleistocene. For these reconstructions, a transfer function (TF) using modern benthic foraminifera surface samples from the Adriatic and Western Mediterranean Seas has been developed. The TF model gives an overall predictive accuracy of ~50 m over a water depth range of ~1200 m. Two separate TF models for shallower and deeper water depth ranges indicate a good predictive accuracy of 9 m for shallower water depths (0–200 m) but far less accuracy of 130 m for deeper water depths (200–1200 m) due to uneven sampling along the water depth gradient. To test the robustness of the TF, we randomly selected modern samples to develop random TFs, showing that the model is robust for water depths between 20 and 850 m while greater water depths are underestimated. We applied the TF to the Pefka E fossil data set. The goodness-of-fit statistics showed that most fossil samples have a poor to extremely poor fit to water depth. We interpret this as a consequence of a lack of modern analogues for the fossil samples and removed all samples with extremely poor fit. To test the robustness and significance of the reconstructions, we compared them to reconstructions from an alternative TF model based on the modern analogue technique and applied the randomization TF test. We found our estimates to be robust and significant at the 95% confidence level, but we also observed that our estimates are strongly overprinted by orbital, precession-driven changes in paleo-productivity and corrected our estimates by filtering out the precession-related component. We compared our corrected record to reconstructions based on a modified plankton/benthos (P/B) ratio, excluding infaunal species, and to stable oxygen isotope data from the same section, as well as to paleo-water depth estimates for the Lindos Bay Formation of other sediment sections of Rhodes. These comparisons indicate that our orbital-corrected reconstructions are reasonable and reflect major tectonic movements of Rhodes during the early Pleistocene.

## 1 Introduction

Since first quantifications of past sea surface temperatures (SST) by Imbrie and Kipp [1], the number of studies that applied transfer functions to microfossil assemblages have increased substantially. Transfer functions (TF) are now widely used to reconstruct paleo-environmental conditions, as for instance past relative sea levels, paleo-SSTs, paleo-precipitation and -salinity [2–10]. The majority of studies which use foraminiferal-based TFs to reconstruct relative paleo-sea levels are restricted to intertidal and shelf areas, and are biased towards younger time intervals such as the Holocene or latest Pleistocene periods. One exception in marine settings is the study of Avnaim-Katav et al. [11], who used a TF to reconstruct relative sea-level changes in the shallow Eastern Mediterranean Sea over a longer time interval, i.e., over the last 1 Myr. However, this study is limited to reconstructions for a few interglacial periods due to the shallow location of the studied sediment cores, lacking suitable glacial sediments. A number of studies estimated changes in paleo-water depths on longer time-scales, e.g., during the Miocene [12, 13] or in the Neogene [14, 15], Neogene to Holocene [16, 17] and early Pleistocene [18]. These studies used different approaches. Some applied semi-quantitative estimates based on water-depth ranges of modern faunas [16, 18] or weighted arithmetic and geometric means of the depth ranges of selected benthic foraminifera to be applied to fossil sequences [12, 13, 17]. Others used the modern analogue technique (MAT; [19]) [14] or MAT in combination with paleo-water depth estimates based on modified plankton/benthos ratios [15].

A prerequisite for accurate paleo-environmental reconstructions is that microfossils have a close relation to the environmental variable of interest [20]. Various studies have shown that benthic foraminifera, particularly in intertidal environments, can be used as accurate relative sea-level indicators [21–28] because they show a strong vertical zonation with respect to elevation in natural intertidal environments [29–31]. This is best explained by the sensitivity of intertidal foraminiferal assemblages to the duration and frequency of tidal flooding and related abiotic factors such as salinity [24, 29, 32]. Other studies indicate that shallow-marine foraminifera can also be used to reconstruct former relative sea levels with different quantitative methods [11, 33–36]. However, in these shallow marine settings (i.e., in the Mediterranean Sea), the foraminiferal distribution is not directly influenced by water depth but by abiotic and biotic factors changing with water depth, in particular by food flux to the sea floor and by substrate type, related to bottom water currents [37–41]. Benthic foraminifera TFs have in contrast never before been used to reconstruct water depths of deeper, i.e., outer shelf to upper slope habitats. This is unfortunate, because here they bear the potential to aid considerably in paleo-water depth reconstructions as water depth (and related gradients in food supply) is likely the major influential factor on the assemblage composition in those deeper environments.

For accurate paleo-environmental reconstructions it is, however, of further importance that the applied TF approach can accurately reproduce the underlying species-environment relationship in a data set. For instance, one often applied method for relative sea level reconstructions is the weighted averaging-partial least squares (WA-PLS) method [42] that is based on unimodal species distribution along the targeted environmental variable. Indirect and direct constrained ordination techniques such as detrended correspondence analysis (DCA, [43]) or detrended canonical correspondence analysis (DCCA, [44]) are widely used to determine whether species have a linear or unimodal response in a data set or along a specific target variable. However, these techniques assume a symmetrical unimodal response shape which is often not the case [45–47]. Alternative approaches can analyze the data directly, by comparing the shape of species distributions with respect to the targeted environmental factor, and determine their response by fitting a reaction curve [48, 49]. Species response curves (coenoclines) towards an environmental factor can be estimated by a variety of methods, including generalized additive models (GAM) and LOESS smoothing [50–53]. These methods have the advantage that the decision is based on real observations of the species response, instead of a derived value as in ordination analyses. Other issues that need to be addressed when using TFs to reconstruct past environmental conditions are unevenly sampled environmental gradients [54], spatial autocorrelation in the data set used to develop the TF [55, 56], the lack of modern analogues [57, 58], and the significance of the reconstructions [59]. Only a thoroughly tested TF can in the end be used to reliably reconstruct the target parameter, by making sure that no other parameters are leaking through or even dominating the derived reconstruction.

**Fig 1:**
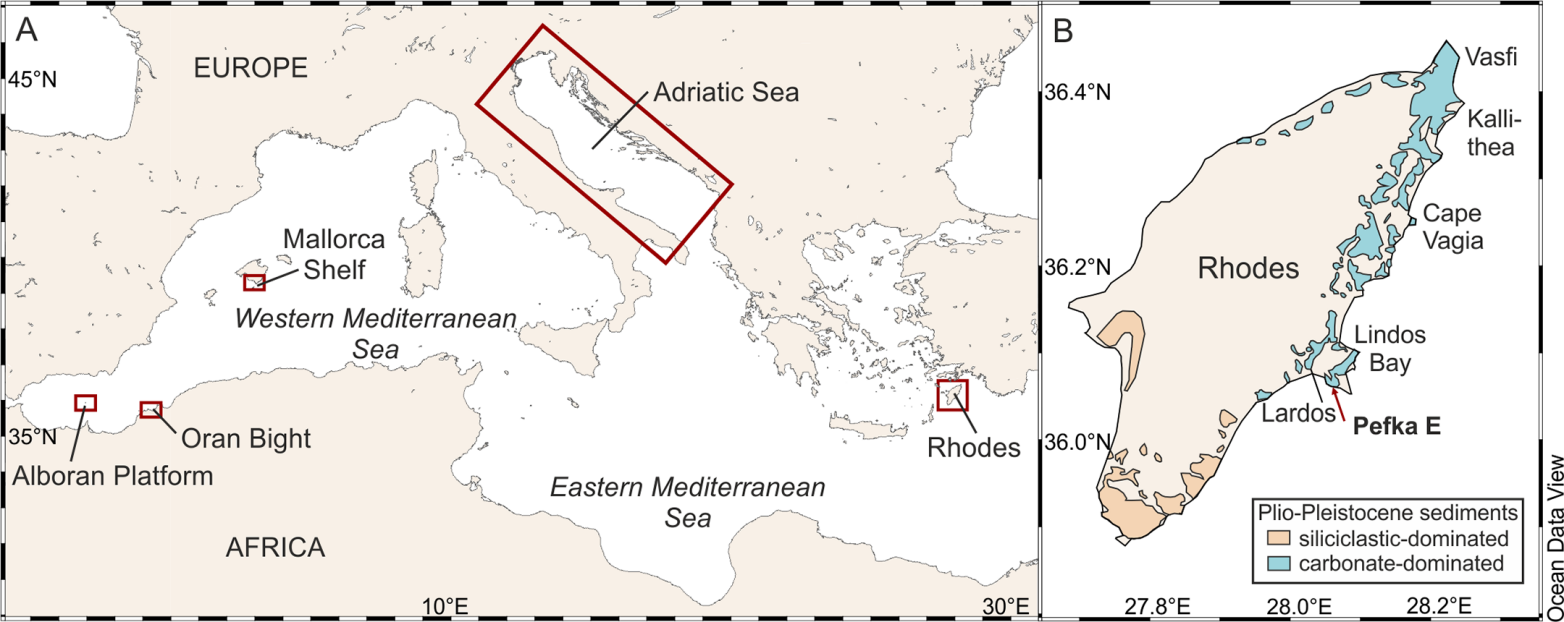
Study area. A. The Mediterranean Sea showing the location of the modern samples (Adriatic Sea [37, 60], Western Mediterranean Sea [61]) used for the development of the transfer function. B. The island of Rhodes with location of the Pefka E section and main Plio- to Pleistocene siliciclastic and carbonate dominated outcrops after [62, 63]. Maps created with Ocean Data View [64].

The major goal of our study is thus to test the applicability of a regional TF based on modern shelf-to-slope foraminiferal assemblages from the Western Mediterranean and the Adriatic Seas to quantify changes in paleo-water depths in an early Pleistocene sediment record from the island of Rhodes (Eastern Mediterranean) (Fig 1). We investigate the Recent species-environment relations and test the accuracy and robustness of our TF that is then applied to the fossil record. We further determine the reliability of the paleo-water depth estimates, and evaluate its usefulness for future quantitative reconstructions.

## 2 Study area

The basement of Rhodes consists of Mesozoic to Cenozoic (Jurassic to Paleocene) rocks, mainly carbonates, which are overlain by terrestrial Mio–Pliocene clastic sediments [62]. The Pliocene clastic fluvial and lacustrine sediments and marine Pleistocene sediments were deposited in isolated basins, especially along the eastern coast of Rhodes [65] (Fig 1B). According to the most recent lithostratigraphic scheme [66], the Plio–Pleistocene deposits are subdivided into three synthems (Trianda, Rhodes and Lindos–Acropolis Synthems), reflecting tectonically-driven transgression–regression cycles over multiple timescales, that are bound by major unconformities. The Lindos Bay Formation (LBF) mainly studied here is part of the Rhodes Synthem that can be subdivided into the transgressive Kolymbia Lindos Bay and St. Paul’s Bay Formations [67], and the regressive Cape Arkhangelos and Tsampika–Ladiko Formations. According to Hanken et al. [63], the up to 20 m thick Kolymbia Formation consists of fossil-rich grain- and packstones with increasing content of terrigenous silt and clay towards the top. The up to 30 m thick Lindos Bay Formation [62, 63] consists of predominantly homogenous (bioturbated) blue–gray marls with a rich microfossil content [18, 63, 68]. Moissette and Spjeldnæs [68] reported an initial increase in water depth followed by a shallowing upwards towards the top of the LBF based on bryozoan zonations. The Cape Arkhangelos Formation consists of a calcarenite that contains bryozoans, mollusks and brachiopods in the lower part, and bivalves and red algae (including large rhodoliths) in the middle and upper part, representing a shallow-marine carbonate environment with a shallowing upward trend [63, 69].

**Fig 2:**
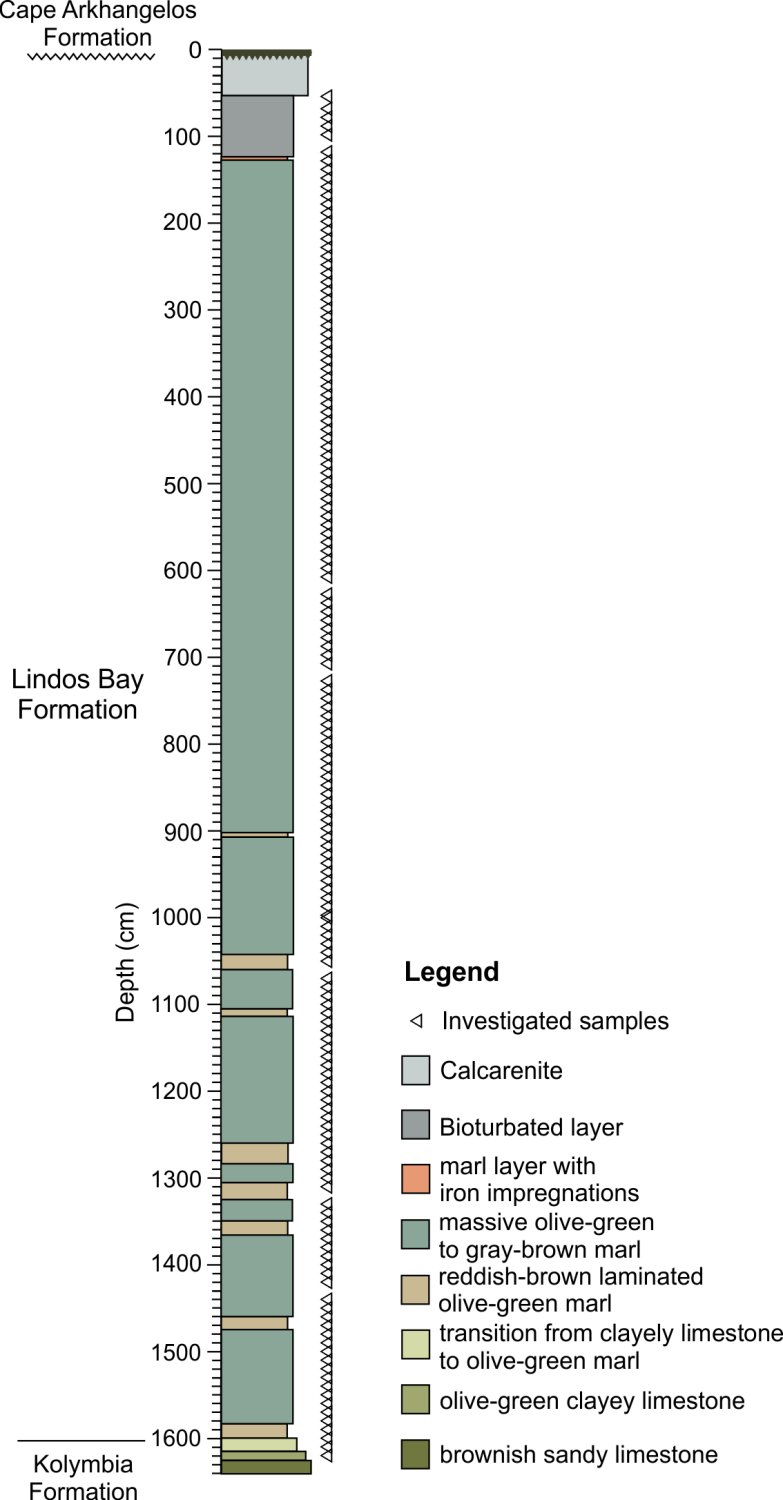
Simplified lithology of the Pefka E section.

The 16.4 m long Pefka E section is situated on the SE coast of Rhodes (36°3’50”N; 28°3’58”E; Fig 1B), 1.5 km south-east and 3.5 km south of the villages Pefka and Lindos, respectively. The section covers the uppermost part of the Kolymbia Formation, the Lindos Bay Formation, and the lowermost part of the Cape Arkhangelos Formation deposited on an erosional surface on top of the LBF (Fig 2). The Kolymbia Formation, at the base of the section (from that the upper 40 cm were sampled), consists of sandy to clayey brown bioclastic limestones. It gradually fines upward into the LBF within 27 cm. The LBF in the Pefka E section has a total thickness of 15.4 m and mainly consists of gray-brown and olive-green homogenous and bioturbated marls with regular intercalations of 5 to 20 cm thick brownish to slightly reddish-gray laminated marl intervals. Within the section, these laminated marl intervals are mainly restricted to the lower and middle parts of the LBF. The upper part of the LBF contains a thin horizon with iron-impregnated cavities and the uppermost 50 cm shows intense bioturbation. The burrows of *Thalassinoides* penetrate the LBF from the erosional unconformity on top of the LBF and are filled with calcarenites of the overlying Cape Arkhangelos Formation.

## 3 Material and methods

### 3.1 Chronostratigraphic framework and stable isotope analysis of the Pefka E section

The chronostratigraphic framework of the Pefka E section is based on a combination of biostratigraphic information and cyclostratigraphy. Biostratigraphic information came from the distribution of calcareous nannofossils. For example, Frydas [70] suggested a deposition within nannoplankton zone NN 19 (see also Titschack et al. [66]), and recently Quillévéré et al. [71] provided a detailed chronostratigraphy of the section, suggesting a deposition from NN 19b to NN 19d based on the highest occurrence (HO) of *Calcidiscus macintyrei*, lowest occurrence (LO) and HO of large *Gephyocapsa*, and HO of *Helicosphaera sellii*. Nannoplankton samples analyzed at 106–109 cm and 1106–1108 cm (from the top) suggest a deposition during nannoplankton zone NN19b based on the occurrence of *Gephyrocapsa aperta*, *Rhabdosphaera claviner*, and *Pseudoemiliana* spp., and the absence of *Discoaster brouweri* and *C*. *macintyrei* (Erlend Martini, pers. comm., 2005). For the cyclostratigraphic framework, we have used the first common occurrence (FCO) of *Hyalinea balthica* (in the >125 μm fraction), which was dated at 1.492 Ma in the Vrica section in Calabria, Italy [72]. The cyclostratigraphic approach is further based on correlation of the filtered time series of the relative abundance of epifaunal species in the >125 μm fraction and the Northern Hemisphere (65°N) summer insolation (June 21) [73]. The relative abundance of epifaunal species was used because they respond sensitively to climate-driven (i.e., precession-driven) changes in oxygen concentrations and food supply at the sea-floor due to sapropel (black shale) formation [74]. Blackman–Tukey spectral analyses of the relative abundance of epifaunal species revealed maxima at 167 and 104 cm, likely representing obliquity and precession cycles and reflecting climate-related changes in trophic conditions. Subsequently, a Gaussian band pass filter, centered at the period of 104 cm, was calculated for comparison with the filtered precessional component (23 kyr cycle) of Northern Hemisphere insolation. Spectral analyses, band pass filtering, and correlations were performed with the software AnalySeries v. 2.0 [75].

Stable oxygen isotope analyses were measured on the epibenthic foraminifer *Cibicidoides pseudoungerianus* with the exception of one sample, where *Cibicidoides pachyderma* s.l. was used due to the lack of *C*. *pseudoungerianus*. Only pristine and clean specimens were picked from the 250–355 μm fraction. We disregarded samples where the calcareous species showed distinct signs of dissolution (i.e., two lowermost samples of the section). The isotopic composition of two to three specimens per sample was measured at the University of Leipzig. The samples were reacted with 105% phosphoric acid at 70°C in a Kiel IV online carbonate preparation device connected to a Finnigan MAT 253 mass spectrometer. Results are given in δ-notation, normalized to the Vienna Pee Dee Belemnite scale (VPDB). External precision (1σ), as determined by replicate analysis of the NBS19 carbonate standard (1 standard per 5 samples), was on average ±0.055‰ for δ^18^O.

### 3.2 Fossil foraminiferal investigations in the Pefka E section

In the years 2000 (lower part of the section) and 2001 (middle and upper parts of the section), 162 samples of 2 cm thickness each were obtained mainly every 10 cm from the 16.4 m long Pefka E section (Fig 1B). Prior to sampling, the weathered surface was thoroughly removed in order to get access to fresh material. All samples were treated with 10% hydrogen peroxide for 24 hours to disaggregate the sediment and were subsequently wet-sieved over a 63 μm screen, dried at 40°C, and then dry-sieved over a 150 μm screen. Foraminiferal investigations were carried out on representative splits containing at least 200–300 benthic individuals of the >150 μm sediment fraction. The >150 μm fraction was used because foraminifera in the modern data used in this study were counted from the same size fraction. Four samples from the Pefka E section (at 617, 1057, 1317, and 1427 cm depths) contained less than 100 specimens per sample, and were thus excluded from further analyses. We further excluded the two lowermost samples (at 1630 and 1640 cm depths) because we noted strong calcite dissolution in these samples, and the six uppermost samples (between 0 and 43 cm depth) because this part of the section is intensely bioturbated and the majority of foraminiferal tests are badly preserved. For the taxonomic identification, preferentially on species level, we used standard illustrations and descriptions [76–80]. When identification on species level was not possible, individual species were grouped into their genus. Potentially reworked specimens (i.e., broken tests, test fragments) were not counted.

### 3.3 Compilation and testing of a combined modern data set for paleo-water depth estimates

For the paleo-water depth estimates, we used two sets of modern surface samples from the Adriatic Sea (269 samples ranging from 8 to 1216 m water depth [37, 60]) and the Western Mediterranean Sea shelf (45 samples ranging from 20 to 235 m water depth, [61]) (Fig 1A). The samples from the Western Mediterranean Sea were re-counted in the >150 μm fraction to ensure compatibility with the Adriatic Sea data set. We compiled these data to cover a wide range of environmental variability in the modern Mediterranean Sea and to ensure that the main fossil species are present in the modern data set. The integration of samples from distant locations further may help to limit the issue of spatial autocorrelation [55, 56]. In the Adriatic Sea dataset, we removed 57 samples with >500 specimens/g sediment, because these samples correspond to lag deposits, as is shown by the large amounts of abraded, broken, and re-crystalized tests. Such samples, in which foraminifera have been strongly concentrated, often contain a mixture of Recent and glacial faunas, so that they are no longer representative of modern conditions and water depth. Next, we excluded all modern samples (*n* = 55) taken from water depths <20 m from the Adriatic Sea data set, because from the fossil faunal composition in the Pefka E section we did not expect to reconstruct paleo-water depths shallower than this. This further helps to reduce the effect of uneven sampling along the water depth gradient, because it eliminates a relatively oversampled depth range [54]. For consistent species assignment of the modern and fossil species, we investigated the individual taxonomic concepts by comparing SEM illustrations and descriptions [37, 60, 80]. The combined modern data set was finally reduced to species with a relative abundance of ≥ 3% in any one sample to exclude very rare species, at the same time ensuring that both data sets contain the majority of abundant species. The modern final data set contains 199 samples and 70 individual taxa (S1 File). In the fossil data set, we excluded all species that are not present in the combined modern data set. This reduced fossil data set contains on average 79% of individuals in the full data set. All datasets (fossil and modern) have been reclosed (i.e., standardized to 100% per sample) using the species that were finally retained in our analyses. In this way, the subcompositional coherence of the datasets is maintained, as is recommended practice for invariable analyses in subcompositional datasets [81].

We applied DCA to the combined modern data to test whether the species have a linear or unimodal response using R (version 3.3.0; [82]) and the R-package “vegan”, version 2.3–3. [83]. We further applied the reaction norm-test written in R (S2 File) to determine the individual species’ response to the water-depth gradient by using four different methods. In all cases, the relationship between the abundance of each species with the environmental factor (here: water depth) is analyzed for its shape (i.e., linear vs. non-linear behavior).

The first method (goodness-of-fit) fits a linear and a quadratic function to the data and tests whether the quadratic function describes the data significantly better, using the *F*-distribution [84], which would imply a non-linear relationship between species abundance and environmental factor. The second method uses piecewise robust linear regression along a moving window to determine the species responses to water depth. Here, the data set is divided into bins of equal size along the range of the environmental parameter, and a robust linear regression based on the M-estimator [85] is applied to every segment. When the slope of the regression line does not significantly differ between all segments, the relationship between abundance and environmental parameter is assumed to be linear along the entire gradient. The third method (binning) calculates mean values of abundance in several bins of equal size along the environmental gradient. When the abundance of the species across all bins either remains constant or else decreases/increases by a constant value from bin to bin (within the range of the 95% confidence interval), a linear relationship can be assumed for this species. The fourth method uses an estimate of the relationship between species abundance and environmental parameter based on a generalized additive model (GAM) on the Gaussian distribution with identity as link function [50, 52]. The Akaike information criterion [86] is used to decide, whether the response curve is linear or non-linear. The four reaction norm test methods have been evaluated using simulated coenoclines which represent truncated or full unimodal as well as linear responses. The tests have shown that the goodness-of-fit and GAM methods are most reliable, with 96.5%, 88.5% and 100%, and 95.5%, 87.0% and 100% correct classifications, respectively, for linear, truncated unimodal and full unimodal behavior. Binning, independent of the number of bins selected, is conservative against a linear response (with only 9.5% correct classifications) and piecewise robust regression is conservative against a truncated non-linear response (with only 21.5% correct classifications) (Table A in S3 File).

As a result of our comparisons between the DCA and reaction norm test results (see section 4.2.1), we applied canonical correspondence analysis (CCA;[44]), with water depth as the only environmental variable, to estimate the amount of variance in the combined modern data set that is explained by the water depth-gradient. We ran additional CCAs on the modern data sets from the Adriatic Sea and the Western Mediterranean Sea to explore species–environment relations in the individual data sets. We again used CCAs for both data sets because DCAs indicate a unimodal species response in both modern data sets (see section 4.2.1). For the Adriatic Sea data set, all species were included into the analysis and water depth, sand content (%), and organic carbon content (%) were used as environmental parameters. For the Western Mediterranean Sea data set, we only used species with a relative abundance of ≥5% in at least one sample. Here, we used water depth, chlorophyll *a* content (mg/m^3^) of the surface water, and substrate (fine-grained sediment <63 μm in per cent) as environmental parameters. Two samples from the Western Mediterranean Sea (samples 352–1 and 393–1) were excluded from the analysis because no grain size data were available for them. All environmental parameters were standardized, and we added binary co-variables to the CCA analysis of the data set from the Western Mediterranean Sea in order to minimize the differences between the three investigated areas [61] (Fig 1). All CCA analyses were performed with the R-package “vegan”.

### 3.4 Development of a regional transfer function for paleo-water depth estimates

For the development of a regional TF, we used WA-PLS. The TF performance was evaluated on the basis of the bootstrapped (1000 cycles) coefficient of determination (*R*^2^_boot_), allowing for a first estimation of the strength of the linear relationship between the observed and estimated water depths in the training data, and the root mean squared error of prediction (RMSEP), allowing for the evaluation of the overall predictive ability of the TF. To avoid model overfitting, a maximum of three components were extracted. The component used eventually was selected according to the lowest RMSEP value if the reduction in prediction error is significant and exceeded 5% for this component compared to the next lower component [42, 87]. Bootstrapping cross-validation (1000 cycles) was also used to evaluate the sample-specific errors of prediction in the fossil data set. We further applied the weighted-averaging (WA) approach to calculate optima and tolerances of the modern species with respect to water depth. For WA-PLS and WA calculations, we used the R-package “rioja”, version 0.9–9 [88].

To test the robustness of our regional TF, we removed randomly selected samples from the modern Adriatic Sea and Western Mediterranean Sea data sets (25% of the samples from each basin) and developed random TFs (*n* = 100) in “rioja” from the remaining modern samples which were then applied to the randomly removed samples.

To determine the individual species’ influence on the reconstructions in the Pefka E section, we applied jackknifing. For this, we reconstructed TFs leaving out one species at a time, and calculated the Kendall rank-order correlation [89] between the final TF model reconstruction and the jackknifed models, as well as the RMSEP of each reconstruction.

### 3.5 Statistically testing the accuracy of paleo-water depth estimates

We applied the goodness-of-fit statistics to evaluate whether the fossil samples from the Pefka E section have a good fit to the water depth in the modern data [57, 58, 90]. We used the 90^th^ and 95^th^ percentiles in the goodness-of-fit statistics as thresholds to differentiate between samples with good (<90), poor (90–95), and very poor (>95) fit to water depth and applied CCA for the ordination. For the calculations, we used the R-package “analogue”, version 0.16–3 [91].

To determine the robustness of our reconstructions, we compared them with reconstructions using an alternative TF method (i.e., MAT). We selected the seven closest analogues in the combined modern data set following the suggestions in Kemp and Telford [90], and used bootstrapping (1000 cycles) to calculate RMSEP and the cross-validated coefficient of determination (*R*^2^_boot_) between observed and estimated water depth in the combined modern data set. For all calculations, we used the R-package “analogue”.

We further tested the significance of our reconstructions by applying the randomTF test [59]. This test calculates the explanatory significance of the TF by comparing its proportion of explained variance with that of many (*n* = 999) alternative models trained on random environmental data, using an ordination technique (e.g., redundancy analysis (RDA); [92]). For the random TF test, we used the R-packages “palaeoSig”, version 1.1–3 [93], “rioja”, and “vegan”. To choose the correct ordination method, we determined whether the species have a linear or unimodal distribution in the fossil data set by using DCA in R package “vegan”.

All data needed to replicate our analyses are available from DRYAD under doi: 10.5061/dryad.349h0.

## 4 Results and discussion

### 4.1 Age model

**Fig 3:**
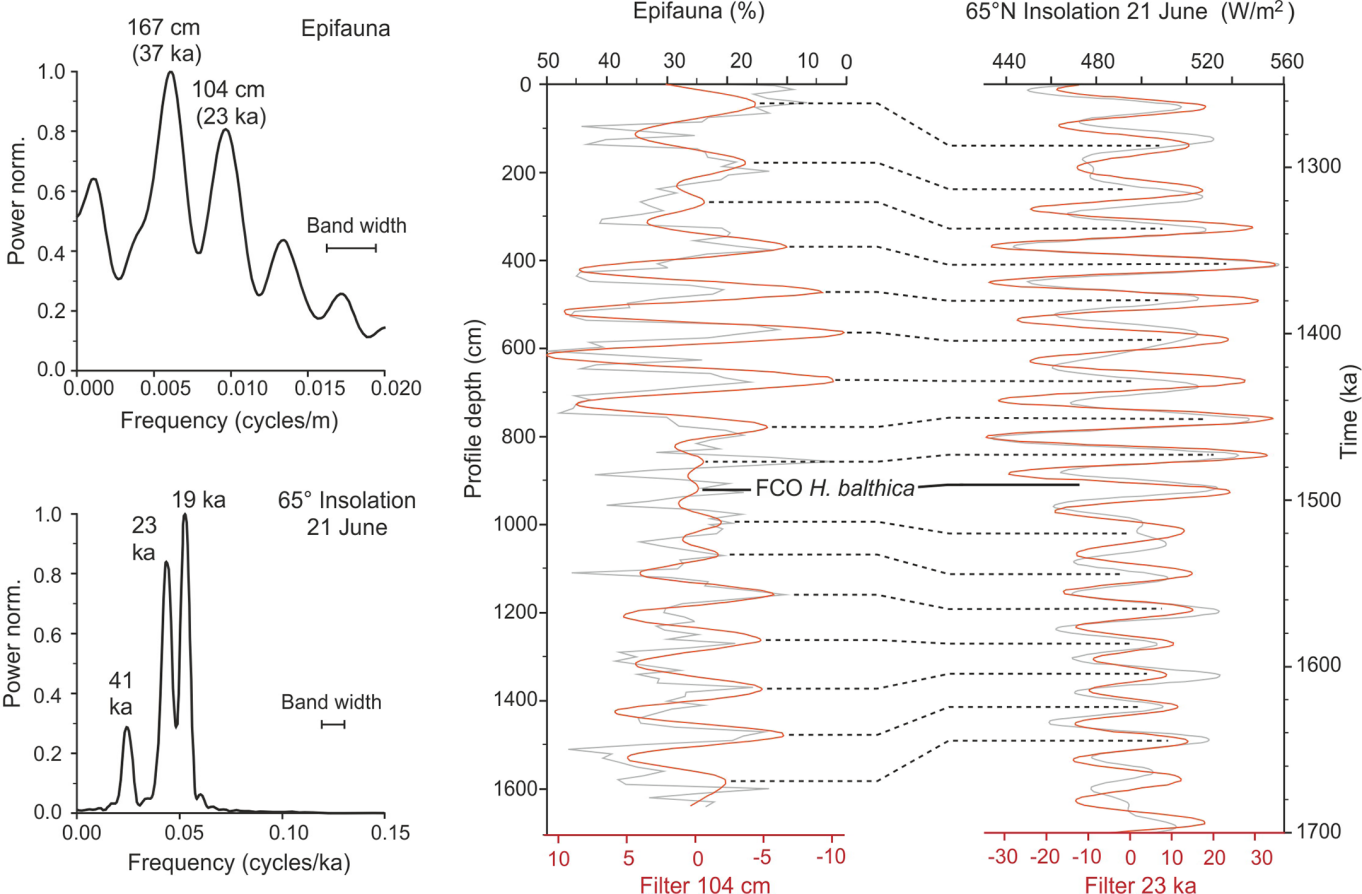
Age model. Results of spectral analysis and Gaussian band pass filtering of the relative abundance of epifaunal species (in the >125 μm fraction) in the Pefka E section and the Northern Hemisphere summer insolation [73]. Maxima of the filtered 102 cm component of relative epifaunal abundance were correlated with the filtered 23 kyr component of Northern Hemisphere summer insolation. The correlation is referenced to the first common occurrence (FCO) of *Hyalinea balthica*, which is dated at 1.492 Ma [72].

**Fig 4:**
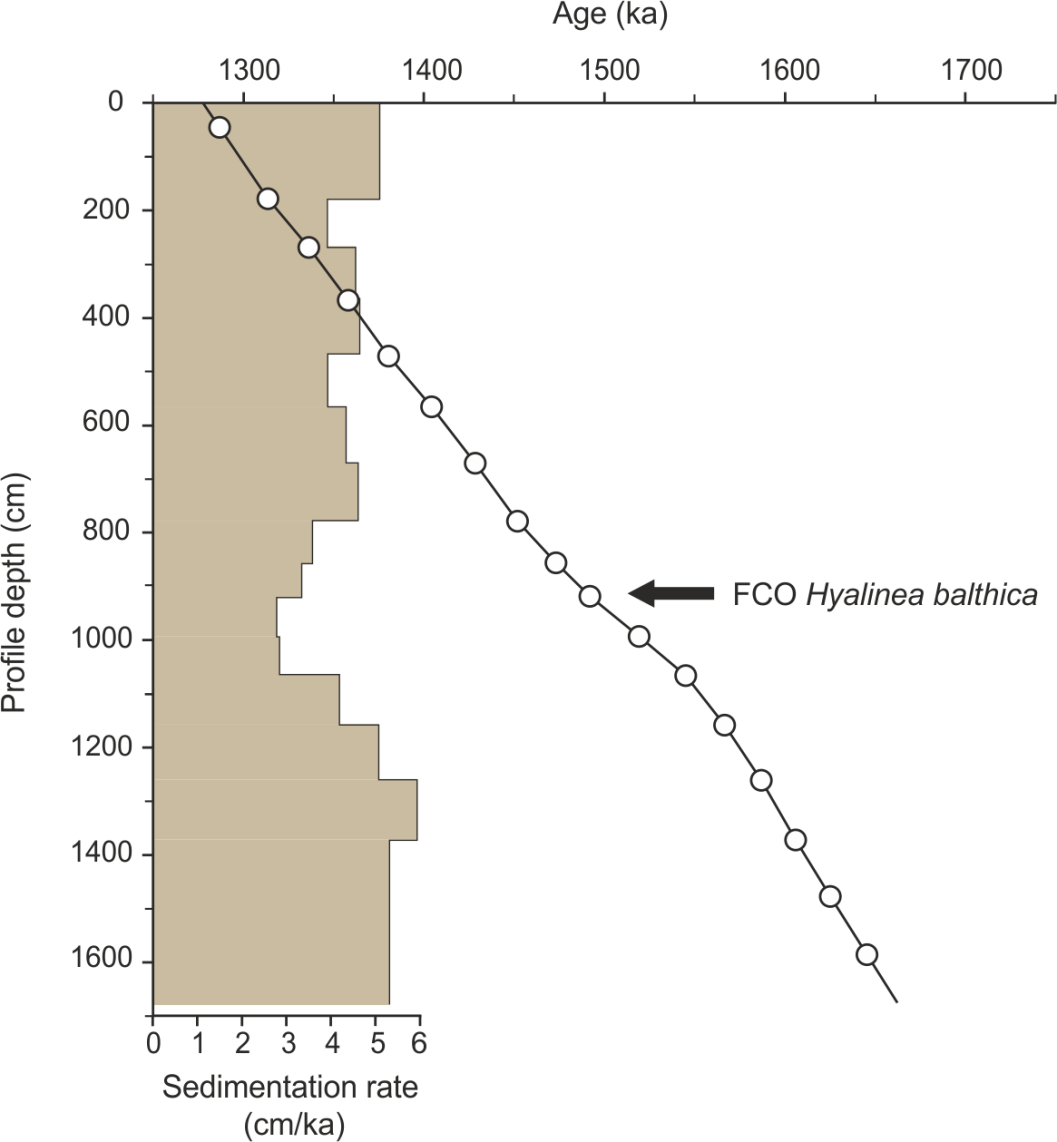
Age-depth plot. Age-depth plot of the Pefka E section with calculated sedimentation rates. Age tie points are derived from the first common occurrence (FCO) of *Hyalinea balthica* and graphical correlation of filtered relative epifaunal abundance and Northern Hemisphere summer insolation. See also Table 1.

At the Pefka E section, the FCO of *H*. *balthica* in the >125 μm fraction was identified at 917 cm section depth representing an age of 1.492 Ma [72] (Table 1). The combined biostratigraphic and cyclostratigraphic framework revealed a depositional age ranging from 1.656 Ma near the base to 1.278 Ma at the top of the LBF suggestion a Calabrian (early Pleistocene) age of the section (Fig 3). Thus, the 16.4 cm thick Pefka E section covers ~387 kyrs. Sedimentation rates range from 2.80 to 5.94 cm kyr^−1^ (average of 4.35 cm kyr^−1^) with generally higher values in the lower part and upper parts and lower values in the middle part of the section (Fig 4). Although we cannot exclude potential errors in peak-to-peak correlations, our age model is in general accordance with available biostratigraphic information on the appearance of calcareous nannofossils (*Gephyrocapsa aperta*, *Rhabdosphaera claviner* and *Pseudoemiliana* spp.) placing the LBF succession of Pefka E into early Pleistocene nannofossil zones MNN19b–d (E. Martini, pers. comm, 2005; [70, 71]). However, the planktonic foraminiferal biostratigraphy revealed considerably older ages of 1.79 to 2.09 Ma for the sediments of the boundary interval between the here investigated LBF and the underlying Kolymbia limestone [71] (Fig 2). This discrepancy may be attributed to reworking of older sediment particles into (both) the Kolymbia limestone and basal LBF leading to overestimated ages for this part of the section.

**Table 1:** Age data used for constructing the age model for the Pefka E section.

Profile depth (cm)	Age (kyrs BP)	Data
46.3	1287.5	Correlation Epifauna–Insolation
179.5	1313.6	Correlation Epifauna–Insolation
268.8	1336.5	Correlation Epifauna–Insolation
369.0	1358.5	Correlation Epifauna–Insolation
471.5	1380.7	Correlation Epifauna–Insolation
565.1	1404.4	Correlation Epifauna–Insolation
671.4	1428.9	Correlation Epifauna–Insolation
777.9	1452.0	Correlation Epifauna–Insolation
856.2	1473.8	Correlation Epifauna–Insolation
917.0	1492.0	FCO *H*. *balthica*
994.1	1519.5	Correlation Epifauna–Insolation
1067.2	1545.1	Correlation Epifauna–Insolation
1158.1	1566.8	Correlation Epifauna–Insolation
1260.9	1587.0	Correlation Epifauna–Insolation
1373.4	1606.0	Correlation Epifauna–Insolation
1476.1	1625.2	Correlation Epifauna–Insolation
1585.9	1645.8	Correlation Epifauna–Insolation

The majority of tie points is derived from correlation of the filtered 102 cm component of the relative abundance of epifaunal species and the filtered 23 kyr component of Northern Hemisphere (65°N) summer insolation (June 21) [73]. The first common occurrence (FCO) age of *Hyalinea balthica* is based on Lourens et al. [72].

### 4.2 Development of the regional transfer function

#### 4.2.1 Response of the species in the modern data set to water depth

The accuracy of a transfer function for paleo-water depth estimates strongly depends on whether or not modern species have a close relation to water depth. To investigate the response of foraminifera in the combined modern data set along the water-depth gradient, we first calculated the length of the gradient along the first axis by using DCA. We measured a gradient length of 4.6 standard deviation (SD) units, which indicates that most species should have a unimodal distribution (Table B in S3 File). However, to determine the individual species response in more detail and along the water depth gradient, we applied our reaction norm-test. The goodness of fit statistics indicates that 57.1% of the modern species have a significant (*p*<0.05) non-linear response to water depth (Table C in S3 File). By using the GAM, 71.4% of the species have a non-linear rather than a linear response to water depth. Piecewise robust regression, using three bins, suggests that only 41.4% of the species have a non-linear response. Binning (three mean bins) indicates 85.7% of the species as having a non-linear response to the water depth gradient, including all of the most dominant species.

**Fig 5:**
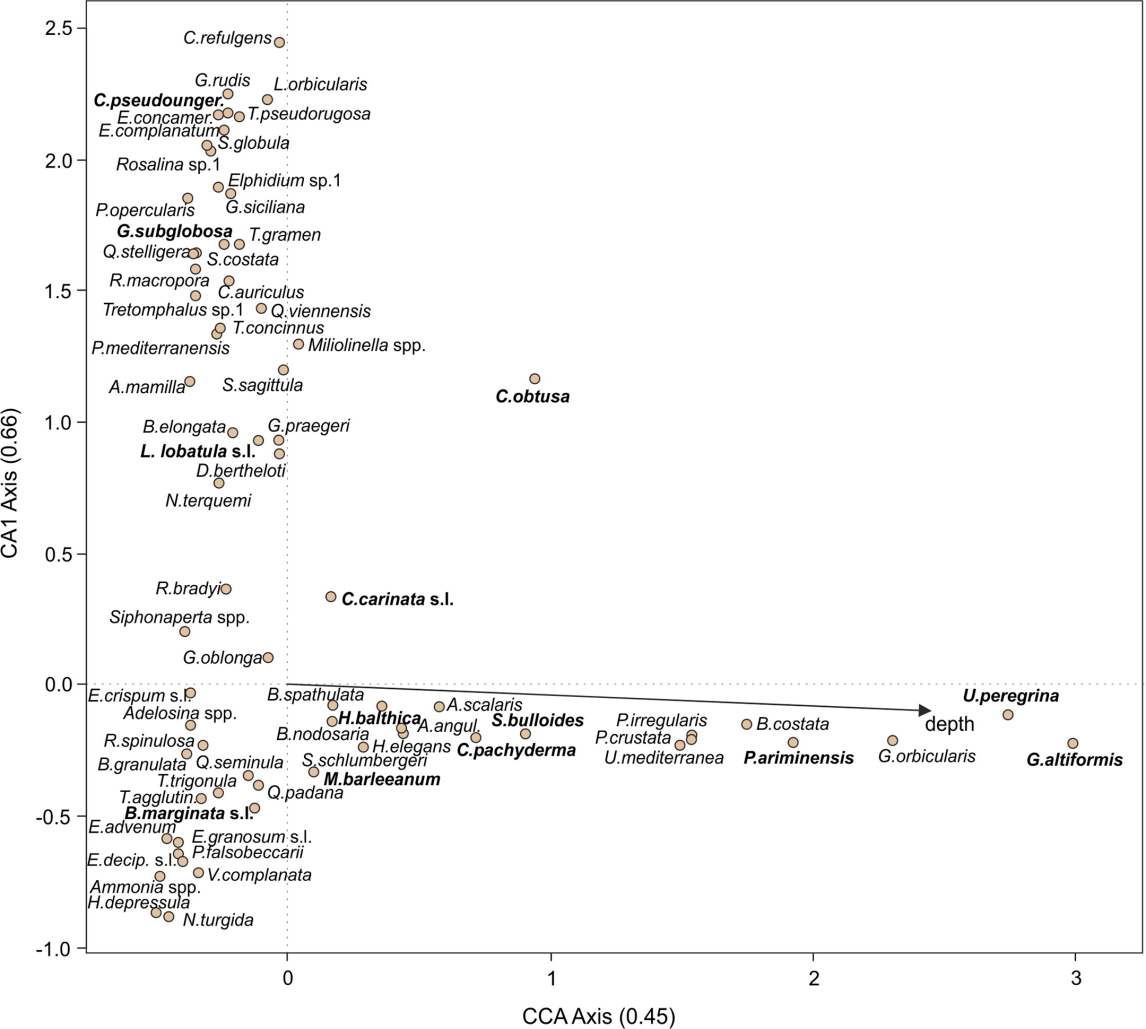
Canonical correspondence analysis (CCA) of the combined modern data set. In bold the species that are dominant in the fossil data set are marked while the eigenvalues of the constrained (CCA) and unconstrained (CA1) axes are given in parentheses.

To determine the amount of variance in the combined modern data set is explained by water depth, we applied CCA, with water depth as the sole environmental variable. The results show that 11.2% of the variance in the combined modern data set is explained by the constrained (first) axis (i.e., water depth), and 16.5% by the second, unconstrained, axis (Table B in S3 File). The water depth gradient is significant with *p*<0.001 (1000 permutations), but the ratio of the eigenvalue of the constraint axis (*λ*1) to the unconstrained axis (*λ*2) is 0.68, indicating that water depth is not the sole ecological gradient in the modern data set [20]. Among the most dominant fossil species in the Pefka E section, and hence important for the water-depth estimates (see section 4.3.1), *Gyroidinoides altiformis*, *Uvigerina peregrina*, *Planulina ariminensis*, *Cibicidoides pachyderma* s.l., *Sphaeroidina bulloides*, and to some extent *Hyalinea balthica* show a close relation to the first axis and thus the water depth gradient. In contrast, other dominant fossil species (*Cibicides pseudoungerianus*, *Globocassidulina subglobosa*, *Cassidulina obtusa*, *Cassidulina carinata* s.l., *Lobatula lobatula* s.l., *Melonis barleeanum* and *Bulimina marginata* s.l.) have a close relation to the second axis (Fig 5). Therefore, the CCA indicates that at least one additional important abiotic parameter influences the modern species distribution. This interpretation is in accordance with earlier observations, where substrate type and food availability (as reflected by total sedimentary organic carbon contents (OC) in the Adriatic Sea and by chlorophyll *a* values reflecting surface production in the Western Mediterranean Sea) have been identified as further important environmental factors [37, 61]. A CCA of the modern data set from the Adriatic Sea shows water depth, ranging between 20 and 1216 m, to be closely related to the first CCA axis explaining 17.5% of the variance in the data set (Table B in S3 File). Sand content and OC are related to the second CCA axis, which explains only 7.4% of the variance in the data set (Fig D and Table B in S3 File). The CCA of the modern data set from the Western Mediterranean Sea shows that water depth, ranging between 20 and 235 m, but also substrate type are the most important parameters for the species distribution. Both are closely related to the first axis, explaining 24.2% of the variance in the data set (Fig E and Table B in S3 File). In the combined modern data set, with water depths ranging between 20 and 1216 m, most of the species which plot on the negative side of the first CA axis are epifaunal species and have their optimum at relatively shallow water depths (Fig 5). This habitat preference suggests the relevance of other environmental factors than water depth, including a close relation to substrate type and bottom water currents, and to a lesser extent to organic carbon fluxes.

#### 4.2.2 Influence of different trophic conditions on the water-depth distribution of benthic foraminifera in the modern Mediterranean Sea

The bathymetric zonations of benthic foraminifera in the modern Mediterranean Sea reveal a characteristic shift from west to east, which can be related to the trophic contrasts between the meso- to eutrophic western basin and the oligotrophic eastern basin [39, 40]. Accordingly, there is a west to east shallowing of the upper or lower depth ranges of most oligotrophic taxa and a decrease or absence of eutrophic (i.e., shallow to deep infaunal) taxa [40]. These observations indicate that organic carbon flux to the seafloor, rather than water depth, is the driving factor for the bathymetric zonation and hence, the western and eastern basins may not be directly comparable due to their different trophic conditions. This raises the question whether or not the environmental conditions in the early Pleistocene were comparable to the modern oligotrophic conditions in the eastern basin. We found shallow to deep infaunal species throughout the section, indicating enhanced organic carbon fluxes to the sea floor. Specifically, we observed between 7% and 80% (on average 34%) infaunal species of which between 4% to 77% (on average 30%) are considered as shallow infaunal (species of the genera *Bulimina*, *Brizalina*, *Cassidulina*, and *Uvigerina*) and between 0% and 19% (on average 3%) as intermediate infaunal (i.e., *M*. *barleeanum*) in the Pefka E section (Fig F in S3 File). Shallow infaunal species, dominated by *C*. *carinata* s.l. and *U*. *peregrina*, are found in high relative abundance throughout the section with a slight decline in the middle part. Intermediate infaunal species are present in low percentages throughout but show a slightly higher abundance in the middle and upper parts of the section.

Some of the dominant fossil species, i.e., *U*. *peregrina*, *C*. *carinata* s.l., and *B*. *marginata* s.s., are supposed to be adapted to fresh organic matter pulses [94–102]. These species have only rarely been observed in the modern Eastern Mediterranean Sea while deep infaunal species are nearly absent [40] except in areas directly influenced by riverine nutrient input and locally enhanced organic matter fluxes [103]. The fossil foraminiferal distributions, i.e., the high relative abundance of infaunal species, suggest at least mesotrophic conditions in the Eastern Mediterranean Sea during the early Pleistocene. This is also supported by Plio- and early Pleistocene foraminiferal assemblages from the Kallithea section in NE Rhodes (Fig 1B; [18]), and by the occurrence of the non-zooxanthellate cold-water corals *Lophelia pertusa* and *Madrepora oculata* in the Lardos section (Fig 1B, [66]). Thus, trophic conditions in the early Pleistocene environments near the island of Rhodes may be better comparable to those of the modern Western Mediterranean Sea, and particularly to those of the modern Adriatic Sea then to the modern environments around Rhodes as also concluded by Rasmussen and Thomsen [18]). More specifically, the modern Adriatic Sea and the Western Mediterranean Sea comprise on average 24% and 8% infaunal species, respectively. Although trophic conditions in the early Pleistocene Eastern Mediterranean Sea and the modern Adriatic Sea (where 77% of the modern data set come from) may to some extent be comparable, we cannot completely exclude bathymetric offsets in the foraminiferal zonations between the modern Western Mediterranean and Adriatic Seas, and between the modern and Pleistocene environments.

#### 4.2.3 Transfer function performance and reliability

**Fig 6:**
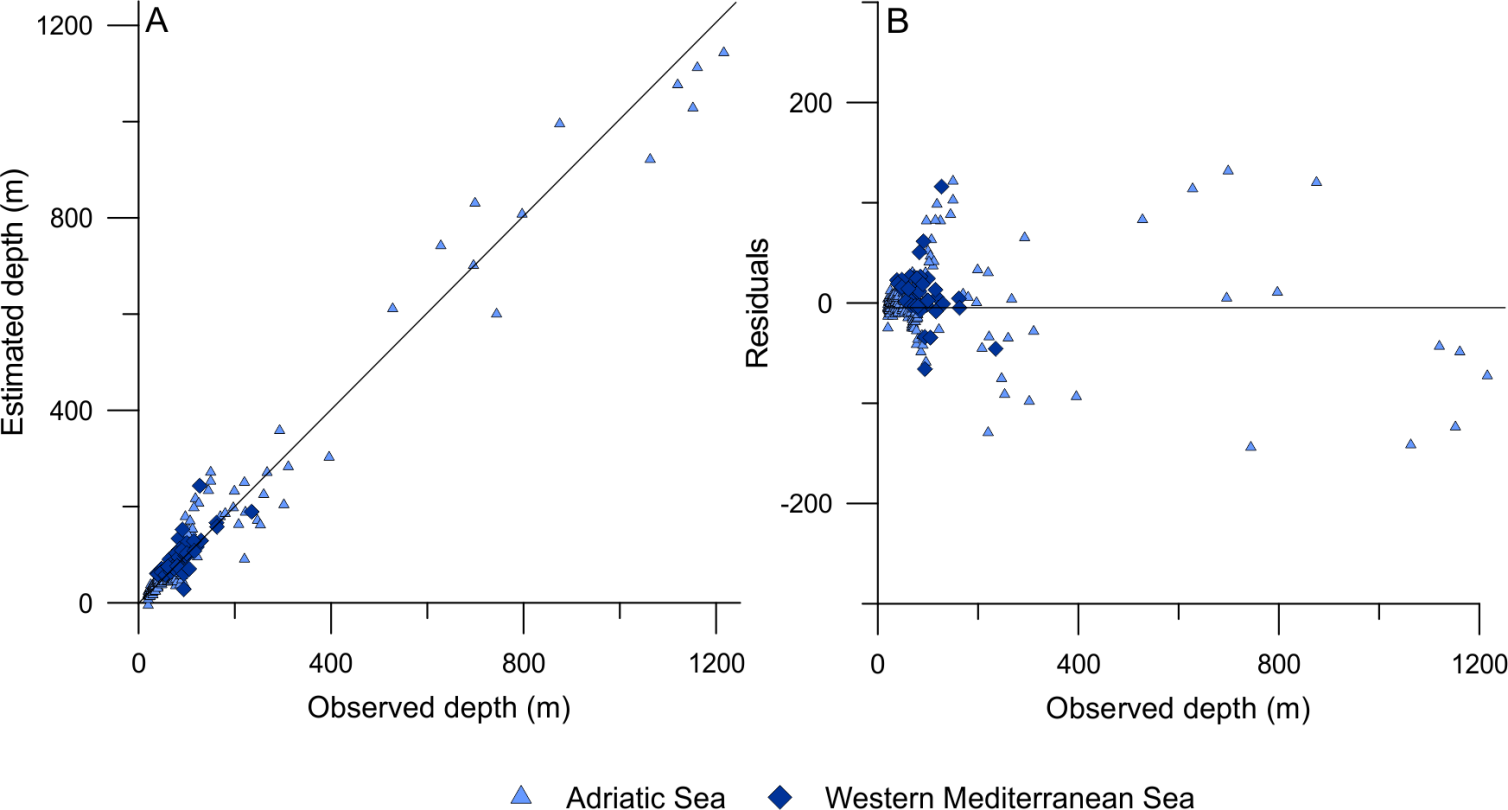
Transfer function performance. Shown are the observed versus estimated water depths (A) and their residuals (B) in the combined modern data set (different colors and symbols are given for the two modern data sets from the Adriatic and Western Mediterranean Seas). See also Table 2.

We ran an initial TF model on the modern data to identify outliers, that is, samples with residuals larger than 2.5 times of the standard deviation of the residuals (5 samples; 2.5%). These samples were then removed from further analysis [104]. For our final TF model, we observed a significant (*p*<0.001) improvement of the RMSEP of 54% from the first component (based on WA with inverse deshrinking) to the second component, and a further significant (*p*<0.001) improvement of 10% from the second to the third component (Table 2). We consequently selected the third component, having a cross-validated coefficient of variation *R*^2^ of 0.95 between the observed and estimated water depths in the combined modern data set and an RMSEP of 49 m, which corresponds to 4% of the observed water-depth range (20–1216 m) in the modern data set. This value is comparable to transfer functions from other intertidal and subtidal settings [11, 34, 35, 87], indicating that our model performs well. However, it should be noted that the RMSEP may be over-optimistic because of the unevenly covered water-depth gradient (i.e., due to the small number of samples from deeper water depths) in the modern data set. To address this issue, we split our data set into two segments (20–200 m with 171 samples and 200–1216 m with 28 samples) and calculated segment-wise RMSEPs as suggested by Telford and Birks [54]. These calculations give RMSEPs of 9 m for the shallower segment but of 130 m for the deeper segment. This shows that the overall predictive accuracy is, in fact, overoptimistic and decreases with increasing water depth. This can be explained by a combination of the low number of samples from larger water depths, and the wider tolerance of deep-water species relative to water depth (i.e., *G*. *altiformis*, *U*. *peregrina*, *Gyroidina orbicularis*, *P*. *ariminensis*, *Bulimina costata*, *Pseudoclavulina crustata*, *Uvigerina mediterranea*, *Pyrgoella irregularis* and *C*. *obtusa*; Fig G in S3 File). The comparison of the observed and estimated water depths in the combined modern data set shows a good fit between both, with residuals ranging from −144 to 132 m water depth (Fig 6).

**Table 2:** Transfer function performance.

	RMSEP	*R*^*2*^_boot_	% change	*p*
Comp01	98.41	0.80	53.70	0.001
Comp02	54.40	0.94	44.71	0.001
**Comp03**	**48.97**	**0.95**	**9.98**	**0.001**

Statistical output of the transfer function performance. Given are the cross-validated root mean squared error of prediction (RMSEP), the cross-validated coefficient of determination (*R*^2^_boot_) between the observed and estimated water depths in the modern data set, the improvement of the RMSEP from one component to the next (% change), and the significance of component fitting increase according to a randomized *t*-test.

In TF models for intertidal environments, commonly the first (WA) component is selected because higher components provide no or only slight model improvements as a result of the dominant influence of marsh elevation (and co-correlated environmental variables) [105–107]. In contrast, TF models for shelf environments showed that higher components commonly yield model improvements due to the influence of more than one environmental variable on the species distribution [11, 34, 35]. The improvement in RMSEP from the first to the second and third components may be the result of the uneven sampled gradient and/or the influence of other environmental parameters. The first WA-PLS component is equivalent to the first axis of a CCA with a single environmental variable [104], and CCA results show that only 11.2% of the variance in the modern data set can be explained by the first axis but 16.5% by the first unconstrained axis (Table B in S3 File). This indicates that additional environmental variables affect the foraminiferal distribution in the combined modern data set. Consequently, the model improvement for the second and third components suggests that WA-PLS, which corrects for edge effects, is also able, to some extent, to exploit the structured pattern in the residuals for higher components after fitting a WA model [42, 104]. However, higher components should not be chosen solely based on the model improvement to avoid a model overfitting [87, 90]. In our case, the selection of the third component seems to be ecologically reasonable because of an obvious influence of additional environmental variables (such as substrate type and food availability) on the modern species distribution (Fig 5, Figs D and E in S3 File).

To test the reliability of our regional TF, we applied 100 randomizations, where the modern dataset was randomly separated into training- (75%) and test-data (25%). We note that there is a correlation between estimated and reconstructed water depths (*τ* = 0.783) based on a Kendall rank-order correlation. The correlations are better for water depths ranging between 20 m and 848 m, but for water depths >848 m all test TFs underestimate the measured water depths (Fig H in S3 File). The performance statistics is almost comparable to the primary TF (Table 2) with mean *R*^2^_boot_ of 0.949 (SD of 0.02) and mean RMSEP of 51 m (SD of 4 m). The component used for the reconstruction was dynamically selected in each randomization according to the criteria described above, and the third component was used in 98 cases. These results imply that our regional TF seems to be robust and can reliably predict water depths as deep as ~850 m, but with a bias towards underestimation beneath that depth.

### 4.3 Application of the regional transfer function

#### 4.3.1 Fossil foraminifera in the Pefka E section

**Fig 7:**
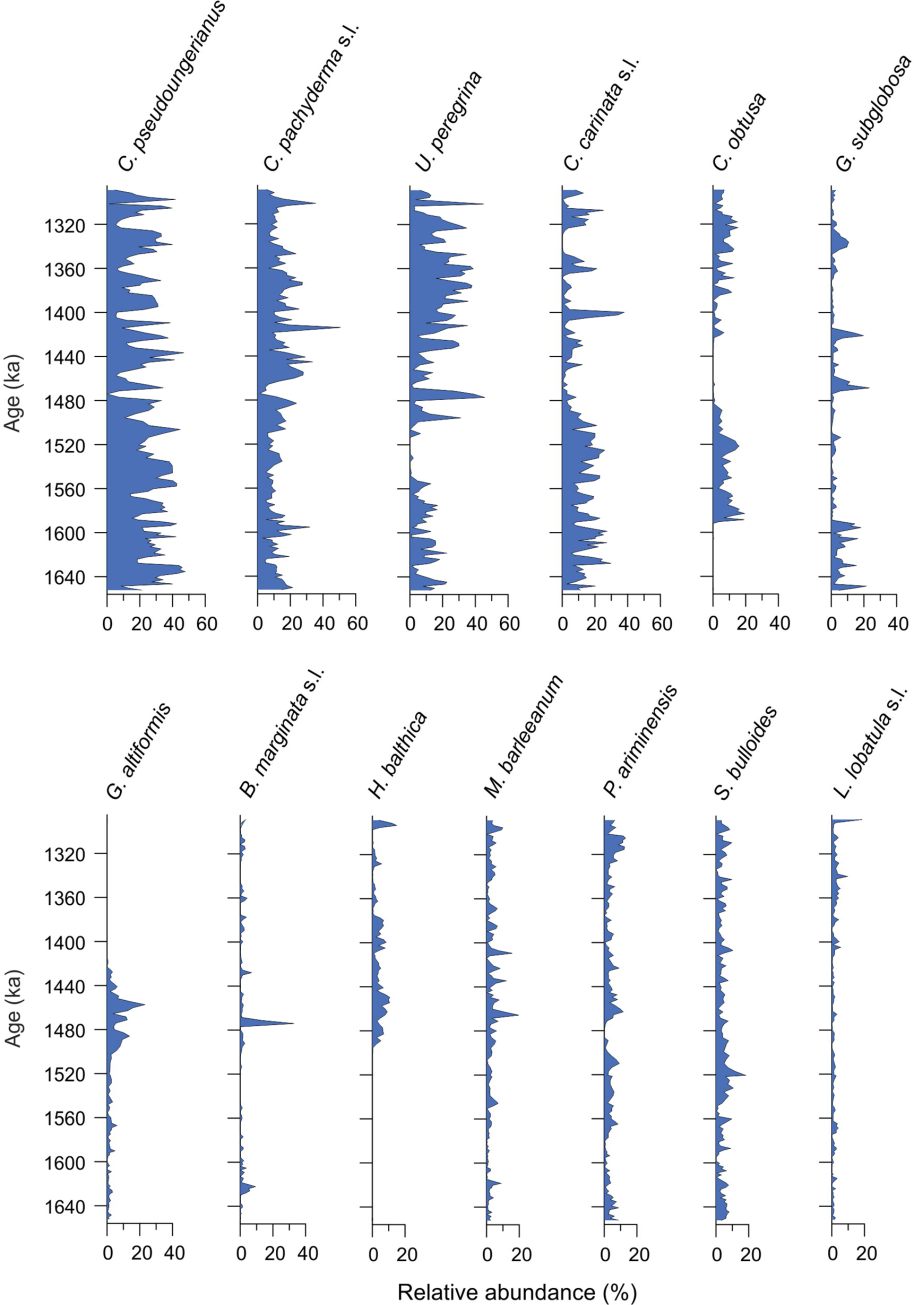
Fossil species of the Pefka E section. Relative abundance of the most important fossil species (≥10% in at least one sample) in the Pefka E section versus age.

A total of 171 different taxa have been identified in the samples from the Pefka E section, with individual samples containing between 29 and 64 taxa. In the data set used for the application of the transfer function, a total of 39 and 28 taxa have an abundance of >1% and >3% in at least three samples, respectively. The most important fossil species (with a relative abundance of ≥10% in at least one sample) in this data set comprise *C*. *pachyderma* s.l. (1–51%), *C*. *pseudoungerianus* (0–48%), *U*. *peregrina* (0–46%), *C*. *carinata* s.l. (0–38%), *B*. *marginata* s.l. (0–33%), *G*. *subglobosa* (0–23%), *G*. *altiformis* (0–23%), *M*. *barleeanum* (0–20%), *C*. *obtusa* (0–19%), *L*. *lobatula* s.l. (0–19%), *S*. *bulloides* (0–18%), *H*. *balthica* (0–15%), and *P*. *ariminensis* (0–13%) (Fig 7). In general, we observed fluctuating relative abundances for the most dominant species in the Pefka E section, but in more detail, *C*. *pachyderma* s.l. and *C*. *pseudoungerianus* are dominant throughout the section with the former increasing in relative abundance towards the top and the latter being slightly more abundant in the lower and middle parts of the section. *Uvigerina peregrina* has a higher relative abundance in the upper part compared to the lower part of the section. *Cassidulina carinata* s.l. has a higher relative abundance in the lower part of the section but it also occurs frequently in the upper part of the section. *Cassidulina obtusa* exhibits two abundance maxima in the middle and upper parts of the section, respectively. *Bulimina marginata* s.l. has a generally low relative abundance except for two samples in the middle part of the section. *Gyroidinoides altiformis* is present in the lower part but is more abundant in the middle part and has not been observed in the upper part of the section. *Globocassidulina subglobosa* is present throughout the section but is more abundant in the lowermost and uppermost parts. *Hyalinea balthica* firstly occurs in the middle part of the section and is then permanently present until the top of the section. *Melonis barleeanum*, *P*. *ariminensis*, and *S*. *bulloides* are present throughout the section. *Lobatula lobatula* s.l. is present in the lower and middle parts of the section but exhibits a higher abundance in the uppermost part of the section.

According to their optimum water depths in the combined modern data set, calculated by weighted averaging, the most important species *G*. *altiformis* (797 m), *U*. *peregrina* (745 m), and *P*. *ariminensis* (560 m) can be considered as indicators for deep water, *C*. *obtusa* (333 m), *S*. *bulloides* (325 m), *C*. *pachyderma* s.l. (288 m), and *H*. *balthica* (208 m) as indicators for intermediate water depths, and *C*. *carinata* s.l. (155 m), *M*. *barleeanum* (152 m), *L*. *lobatula* s.l. (106 m), *B*. *marginata* s.l. (104 m), *G*. *subglobosa* (98 m), and *C*. *pseudoungerianus* (88 m) as indicators for shallow water depths (Fig G in S3 File). Although we probably do not cover the complete water depth range of some of the most important species in the fossil record, our interpretations are supported by a number of observations from the Mediterranean Sea [16, 38–40, 76, 102, 108, 109].

#### 4.3.2 Determining the robustness and accuracy of the paleo-water depth estimates in the Pefka E section

**Fig 8:**
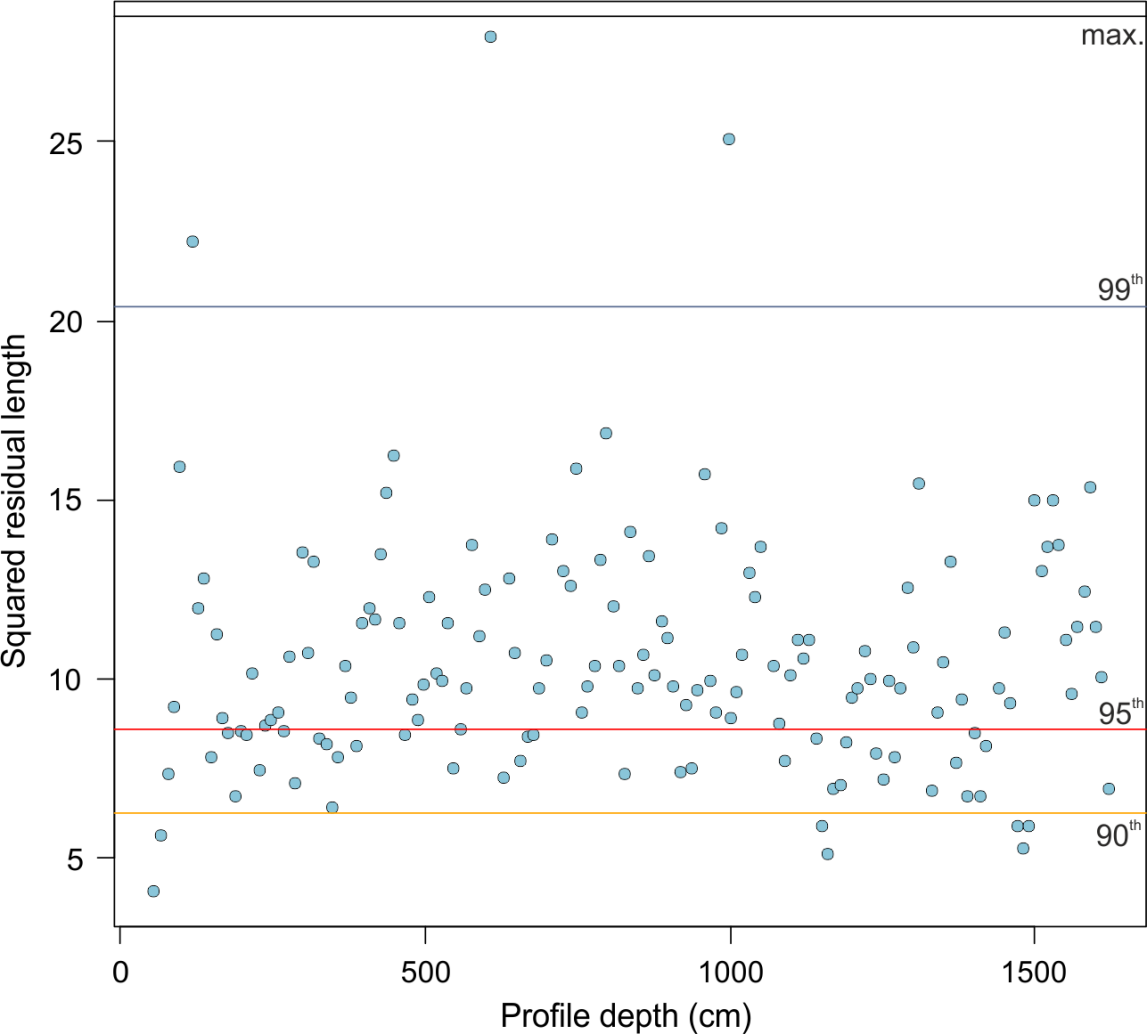
Goodness-of-fit statistics. Squared residual length of the fossil samples from the Pefka E section versus depth is shown. The 90^th^, 95^th^, and 99^th^ percentile and the maximum residual length are indicated with lines.

The accuracy of paleo-environmental reconstructions depends not only on the species response to the target environmental variable but also on the availability of modern analogues for fossil faunas and the fit of the fossil species to water depth. To explore whether or not our fossil samples have a good fit to water depth, we applied the goodness-of-fit statistics [57, 58]. The goodness-of-fit test indicates that only 7 fossil samples (5%) have a good fit, 39 samples (26%) have a poor fit, but the majority of the fossil samples have a very poor (103 samples; 68.0%) or extremely poor (>99, 3 samples; 2%) fit to water depth (Fig 8). These results are intriguing, and are investigated in more detail in the following. A coverage plot of the maximum relative abundance of individual species in the modern and fossil data sets illustrates that a number of species have a higher relative abundance in the fossil compared to the modern data set (Fig I in S3 File). Among these, the most dominant (and thus most important) species in the Pefka E section have a higher maximum relative abundance (between 38% and 51%) but a lower maximum relative abundance (of 16% to 30%) in the modern data set. We therefore assume that the poor to very poor fit to water depth in the Pefka E fossil data set is the consequence of a lack of close analogues in the modern data, indicating that local paleo-environmental conditions (i.e., productivity) during the early Pleistocene perhaps were less comparable to the modern conditions than previously thought (see 4.2.2). A number of tests with simulated data showed, however, that WA-PLS performs well under mild analogue situations [110, 111]. Also, Hutson [112] tested how well different transfer function techniques work under non-analogue situations and found a technique based on weighted averaging to be most accurate, because it does not extrapolate as other techniques do.

To determine the individual influence of the species on the paleo-water depth estimates, we ran additional TFs by excluding species by jackknifing and compared the reconstructions of the derived TFs with our final model by calculating Kendall’s rank-order correlation and RMSEP. These results show that the exclusion of *U*. *peregrina* resulted in the lowest correlation (*τ* = 0.08) and highest RMSEP (278 m) in the Pefka E section, indicating that this species has the strongest influence on the estimates (Fig J in S3 File). Further species with a strong influence on the reconstructions (higher RMSEP) comprise *C*. *pseudoungerianus* (118 m; *τ* = 0.836), *C*. *pachyderma* s.l. (104 m; *τ* = 0.888), *C*. *carinata* s.l. (61 m; *τ* = 0.847), and *G*. *altiformis* (61 m; *τ* = 0.846) (Fig J in S3 File).

The species with a higher relative abundance in the fossil record compared to the modern data sets and/or with a strong influence on the estimations have their optimum modern water depths at 88 m (*C*. *pseudoungerianus*), 155 m (*C*. *carinata* s.l.), 288 m (*C*. *pachyderma* s.l.), 745 m (*U*. *peregrina*), and 797 m (*G*. *altiformis*) (Fig G in S3 File). Accordingly, the TF may overestimate paleo-water depths for samples strongly dominated by *U*. *peregrina* and *G*. *altiformis* and may underestimate paleo-water depths for samples strongly dominated by *C*. *pseudoungerianus* and *C*. *carinata* s.l. As a consequence, we screened the three samples with an extremely poor fit to water depth (squared residual lengths larger than the 99^th^ percentile). Two of the samples are strongly dominated by both *U*. *peregrina* (45% and 23%) and *C*. *pachyderma* s.l. (35% and 50%), respectively. The third sample has a high relative abundance of *S*. *bulloides* (18%). We finally removed these three samples from the reconstructions.

To determine the robustness of TF reconstructions, it is recommended to compare different TF techniques [90, 104]. We therefore compared our paleo-water depth reconstructions based on the WA-PLS method to those using MAT. We found a cross-validated correlation between observed and predicted water depths in the combined modern data set of *R*^*2*^ = 0.98 and a RMSEP of 55 m for MAT, indicating a performance comparable to WA-PLS (compare Table 2). The reconstructions in the Pefka E section are very similar for both models (*r* = 0.77; Fig K in S3 File). This indicates that our reconstructions seem to be reasonable.

**Fig 9:**
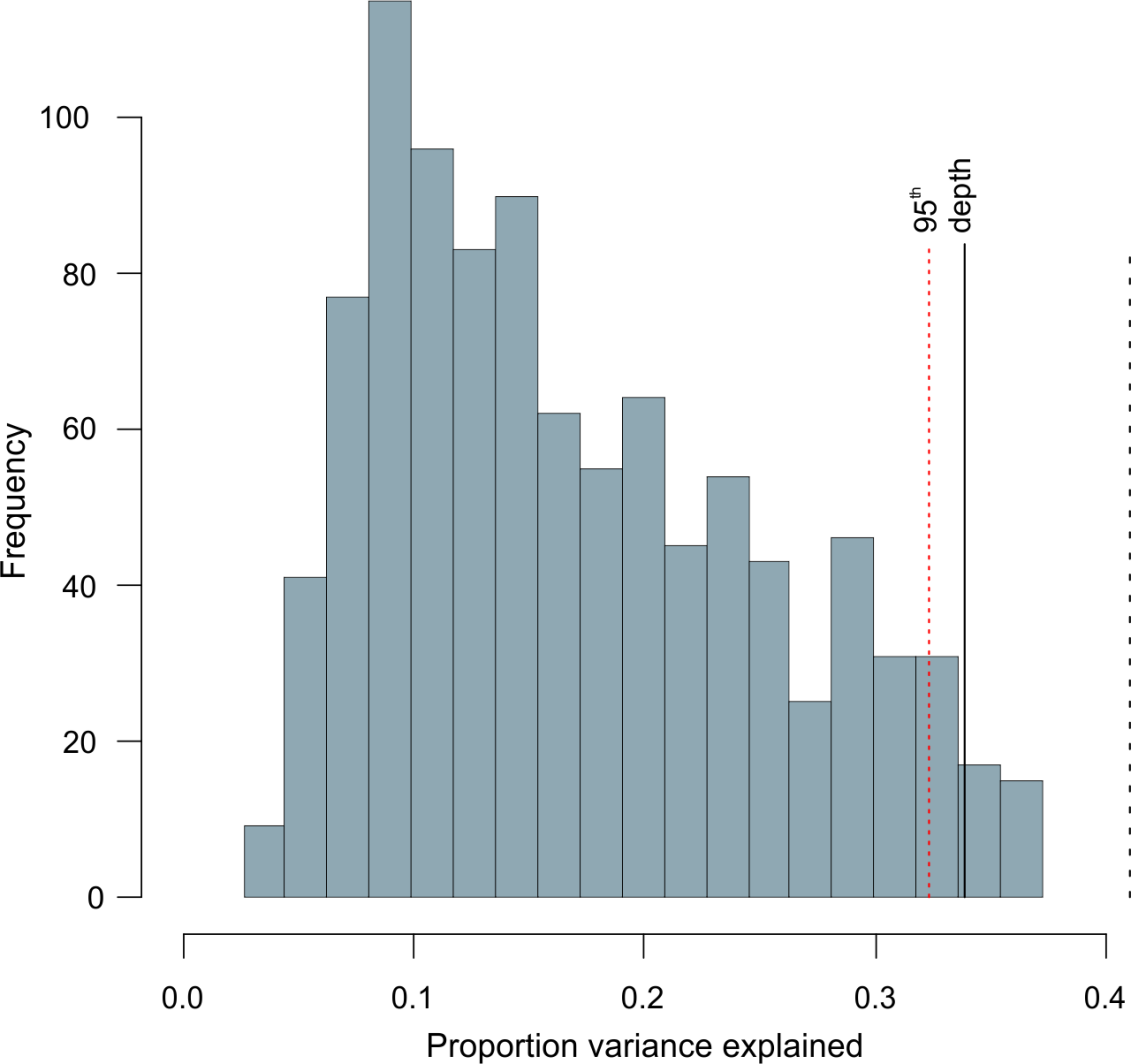
RandomTF test results. Histogram of the proportion of variance in the fossil data set explained by 999 transfer functions trained with random data, and the proportion of variance explained by the transfer function constructed by us (solid black line). Dotted red line shows the 95%-explained limit and dashed line the maximum variance in the fossil data set.

Recent studies [59, 113] highlighted the necessity to test the significance of paleo-environmental reconstructions when using microfossil-based TFs. We therefore applied the randomTF test [59] to test the significance of our reconstruction. We used RDA for ordination because of the relative short gradient length (2.15 SD units) along the first axis, indicating a more linear rather than unimodal species distribution in the fossil data set (Table B in S3 File). We ran the test to the data set excluding the three fossil samples with extremely poor fit to water depth as described above. The result shows that our paleo-water depth reconstructions are significant at the 95% confidence level (Fig 9), confirming that our reconstructions seem to be robust [59].

### 4.4 Paleo-water depth trends in the Pefka E section and comparison with other records

**Fig 10:**
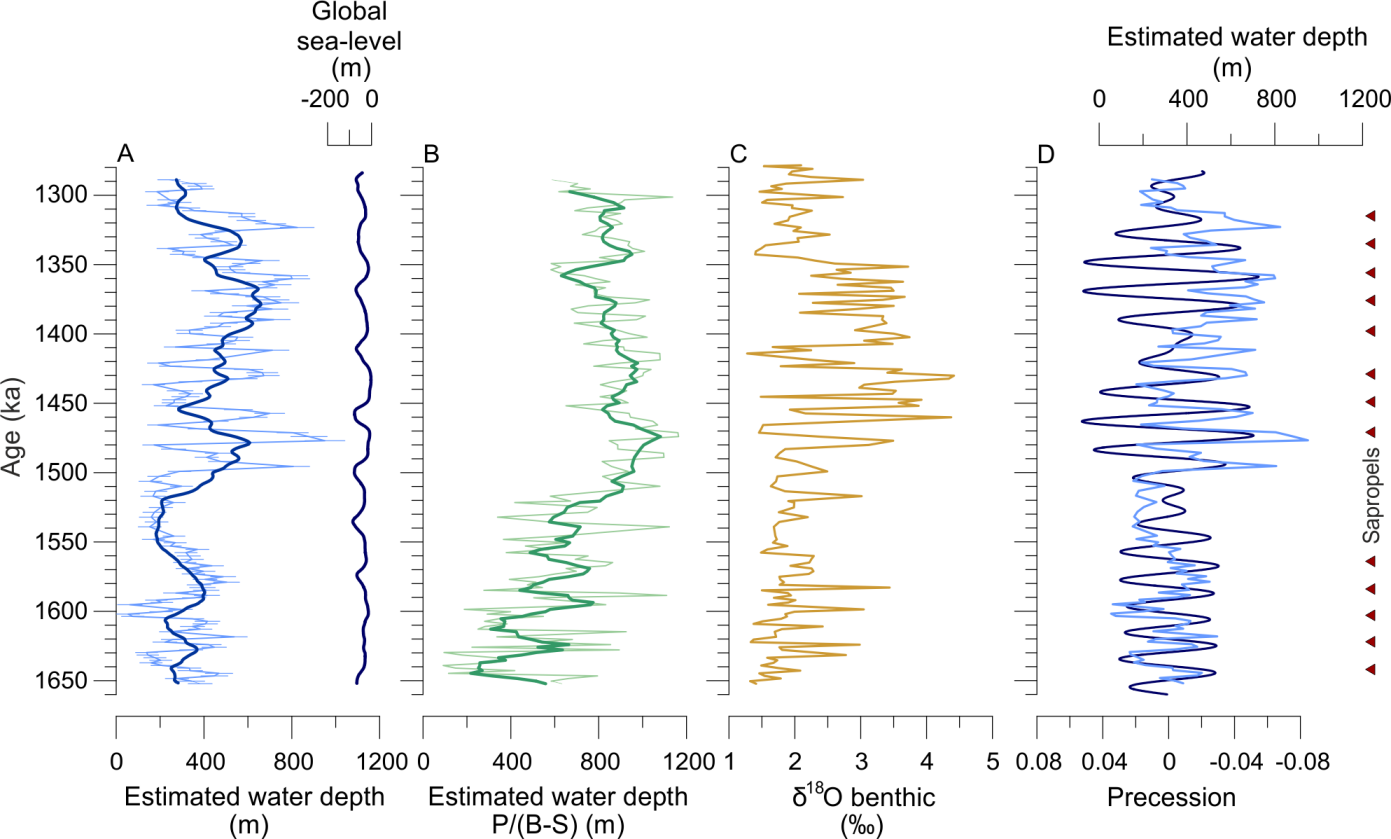
Paleo-water depth reconstructions for the Pefka E section. A. Original paleo-water depth reconstructions (excluding those samples with extremely poor fit to water depth) as thin line with sample specific error (error bars) and moving average of paleo-water depths with window size matching average precession period. For reference, global ice volume transferred into global sea level in meter relative to present [125] is given. B. Paleo-water depth estimates based on planktic/(benthic−stress markers) (P/(B−S)) ratios using the transfer function from Van der Zwaan et al. [123], with the thin green line showing the original reconstruction and the thick green line showing a five point moving average. C. Benthic foraminifera stable oxygen isotope ratios (δ^18^O). D. Comparison of the original paleo-water depth reconstructions (light blue) versus precession (dark blue) [73]; note inverse scaling of the precession curve. Symbols show Pleistocene sapropels identified in ODP Site 967 and ODP Hole 969D (Eastern Mediterranean Sea) [72].

To determine the reliability of our paleo-water depth reconstructions, we here want to compare them to other records available from the island of Rhodes. In general, our paleo-water depth reconstructions show a long-term transgressive trend, followed by a regressive phase in the upper part of the section, which may reflect tectonic movements of the island of Rhodes during the early Pleistocene (Fig 10A). However, the reconstructions also show distinct and repeating short-scale fluctuations with amplitudes of up to around 800 m, particularly in the middle part of the section. These abrupt fluctuations cannot be explained by tectonic adjustments but may represent a bias linked to orbital-driven environmental changes and resulting bathymetric species shifts. The Neogene climate and hydrology of the Mediterranean Sea was strongly influenced by changes in precessional insolation, resulting in cyclic depositions of sapropels (black shales), particularly in the eastern basins [72, 114–119] (Fig 10D). The sapropels were formed during Northern Hemisphere summer insolation maxima/high-amplitude precession minima, resulting in an enhanced African monsoon [120], and as a consequence, in higher freshwater influx, particularly via the Nile River, and higher export productivity into the Mediterranean when compared to today (review in Rohling et al. [74]). To determine whether or not these orbital-driven changes in hydrology and paleo-productivity (Fig 10D) may have an influence on our reconstructions, we evaluated our reconstructions and orbital precession [73] for multicollinearity [121]. We allowed to find the optimal time lag between both records and found a slight but significant Pearson product-moment correlation between both records with *r* = 0.271 and *p*<0.001, suggesting that changes in the precession band indeed influence our reconstructions. This is not surprising because infaunal species are abundant and show marked fluctuations throughout the Pefka E section, suggesting a response to precession-related changes in paleo-productivity (Fig F in S3 File). For example, the most dominant species *U*. *peregrina* in the middle and upper parts of the Pefka E section has been found in high relative abundance in a middle to late Holocene sediment record, reflecting enhanced seasonal phytodetritus fluxes related to Nile river nutrient plumes [103]. As a consequence, we applied a moving average smoothing using a calculated average precession period of 20.5 kyrs to filter out the precession-driven component from our reconstructions (Fig 10A). The precession-corrected paleo-water depth estimates reveal two marked transgressive and regressive trends with water depth changes of 190 to 410 m between 1652 and 1546 ka, followed by relatively stable paleo-water depths of around 190 m between 1544 and 1531 ka, and two distinct transgressive phases with maximum paleo-water depths of 610 and 650 m, separated by one short-time regression phase with a minimum paleo-water depth of 290 m between 1531 and 1369 ka (Fig 10A). The upper part of the section is characterized by two regressive phases with minimum paleo-water depths of 417 and 249 m between 1368 and 1316 ka, separated by a short-term transgressive phase with a maximum paleo-water depth of 560 m. Paleo-water depths then slightly fluctuate between 250 and 340 m between 1316 and 1285 ka.

By using the smoothed paleo-water depth curve as a basis, we compared our estimates to paleo-water depth estimates based on the relative abundance of planktonic foraminifera on the total foraminiferal fauna (P/B ratio) counted in the >125 μm fraction of the Pefka E section. Specifically, we used a modified ratio by excluding infaunal species (stress markers S) as described by van Hinsbergen et al. [122] and applied the formula given in Van der Zwaan et al. [123] (Fig 10B). The paleo-water depths (smoothed using a five-point moving average) based on the P/(B−S) ratios fluctuate between 220 and 780 m from 1652 to 1530 ka, suggesting a general increase in paleo-water depth, between 600 and 1080 m from 1527 to 1360 ka, and between 630 and 950 m from 1359 to 1297 ka. It has been shown that paleo-water depth estimates based on P/B ratios do not always give reasonable quantitative results due to specific local environmental conditions such as exposure to the open ocean, surface currents and trophic conditions [124]. However, for the Pefka E section the long-term transgressive and regressive trends of our TF based reconstructions are also reflected by the reconstructions based on the P/(B−S) ratios although absolute values of the P/(B−S) ratios are generally higher.

During the time of the deposition of the Pefka E section, orbital-scale eustatic sea-level changes can account for a maximum fluctuation of 85 m (Fig 10A; [125, 126]), suggesting that the long-term and corrected relative sea-level trend observed in the Pefka E section, with a maximum difference of about 470 m, is caused by substantial local to regional vertical tectonic movements, particularly between 1531 and 1316 ka. This interpretation is supported by the epibenthic δ^18^O record of this section that basically retraces the long-term relative sea-level trend (Fig 10C). The δ^18^O values range between 1.32 and 3.44 ‰ (on average 1.90 ‰) from 1620 to 1481 ka, and reveal an increase with maximum values of 3.72 and 4.42 ‰ (average 2.95 ‰) from 1478 to 1351 ka, followed by a decrease to values between 1.40 and 3.04 ‰ (average 1.95 ‰) from 1349 to 1271 ka. Shifts to higher water depths are accompanied by calcification under lower ambient bottom water temperatures. In the modern Eastern Mediterranean Sea, there is a decrease in temperature of ~0.5–0.7°C per 100 m [127] which would account for 0.8 to 1 ‰ in δ^18^O when paleo-water depth increased by ~400 m [128]. Additional effects of global ice-volume changes of around 1 ‰ for a maximum global sea-level change of 85 m during the deposition of the section [125] and potential salinity changes of Eastern Mediterranean surface and intermediate waters [129, 130] add to the temperature influence and likely account for the high-amplitude δ^18^O shifts of more than 2 ‰, particularly in the middle and upper parts of the section. However, it should be noted that we cannot exclude the potential influence of diagenetic effects on the stable isotope signal although the measured tests did not exhibit visible signs of dissolution and presence of cements.

For further regional evaluations of our reconstructed relative sea-level history for the Pefka E section, we compared the range of our corrected estimates with reported paleo-water depth ranges from other sediment sections on the island of Rhodes. According to Hanken et al. [63], the Kolymbia Formation reflects a deepening upward indicated by shallow-water benthic foraminiferal faunas (dominated by *Elphidium* spp. and *Ammonia* spp.) in the lower part and deeper water assemblages with presence of *Uvigerina* spp. and *Bolivina* spp. in the uppermost part of the formation. This deepening is also reflected by ostracod faunas, which revealed water depths of >250 m at the boundary between the Kolymbia Limestone and the LBF (Hanken et al. [63], and references therein). These values are in accordance with our estimates of 260 to 290 m for the lowermost part of the LBF. Moissette and Spjeldnæs [68] reported an increase in water depth across the LBF, mainly based on bryozoan zonations, with water depths ranging between 250 to 450 m and a maximum value of ~600 m for the Lindos Bay, Vasfi and Cape Vagia sections (Fig 1B). Finally, water depths ranging between 320 and 500 m based on benthic foraminiferal and ostracod assemblages for the LBF deposition at the Kallithea section in the NE part of Rhodes were reported [18, 131] (Fig 1B). These observations are in general accordance to our estimates, indicating a deepening with maximum water depths of 610 to 650 m in the middle to upper part of the section.

Although it is not the primary focus of our study, the reconstructed bathymetry of the Pefka E section presents some important implications for the regional development of the island: (i) When comparing the onset of the LBF deposition at Pefka E section with other localities [66], and especially the new chronostratigraphy of some sections provided by Quillévéré et al. [71], the onset of the deposition seems rather synchronous in the southern part of the island. Only at Kalithea the onset of LBF deposition potentially occurred subsequent to the phase of maximum transgression within the Pefka E section [132] (compare with Cornée et al. [133] for chronostratigraphic inconsistencies). This might imply a differential paleo-bathymetric development of northern Rhodes compared to central and southern Rhodes. (ii) The phase of maximum transgression within the Pefka E section between 1.5 and 1.35 Ma might imply that the it was either reached earlier than suggested by Titschack et al. [66] or Cornée et al. [133], or that it lasted much longer (until about 900 ka) and was interrupted by a short phase of uplift (represented in the upper part of the Pefka E section). The regressive phase during LBF deposition, reflecting sustained uplift of Rhodes, is preserved in the Lardos section (Fig 1B) where a shallowing from ~320 to <100 m water depth towards the top of the LBF has been observed [66].

The good agreement of the long-term water depth trend for the Pefka E section with available semi-quantitative reconstructions for other sediment sections of Rhodes confirms the general reliability of our reconstructions. Accordingly, we provide a first quantitative reconstruction of long-term relative sea-level changes during the deposition of the LBF. Our results reflect substantial local to regional vertical tectonic movements during the deposition of the LBF and provide information on the exact timing of neotectonic events for the island of Rhodes.

## Conclusions

We developed a regional TF model based on benthic foraminiferal assemblages, covering a wider water depth range than any other model before. For this, we used a combined data set of modern shelf to slope samples from the Western Mediterranean and Adriatic Seas. We found a relation of the modern assemblages to water depth but also to other factors such as substrate and food availability. For the developed TF, we observed a predictive accuracy (RMSEP) of about ~50 m over a water depth range of ~1200 m which is comparable to other TF performances. However, it should be noted that segment-wise RMSEPs indicate a better predictive accuracy of 9 m for shallower water depths (0–200 m) but a worse predictive accuracy of 130 m for deeper water depths (200–1216 m). By applying a randomization test, we found our model to be robust for water depths ranging between 20 and 848 m, indicating that paleo-water depth reconstructions within this range can be reliable if the assemblages are not strongly influenced by other environmental parameters. We applied the model to the early Pleistocene Pefka E sediment section situated in the SE of the island of Rhodes and tested whether the fossil species have a good fit to water depth in the modern data set. We observed that most fossil samples have a poor to very poor fit to water depth which can be explained by the lack of close modern analogues. We screened the fossil samples, resulting in exclusion of samples with extremely poor fit to water depth, and determined whether or not the reconstructions are robust by comparing with an alternative TF model (i.e., based on the modern analogue technique). This comparison showed that both reconstructions are comparable. Although a randomization transfer function (randomTF) test revealed that our reconstructions are significant at the 95% confidence level, and hence appear generally trustworthy, we found our reconstructions to be strongly influenced by precession-related changes in paleo-productivity in the Eastern Mediterranean Sea and consequently filtered out this precession component. Our precession-corrected paleo-water depth estimates are in general accordance with reconstructions based on modified P/B ratios and epibenthic δ^18^O data of the same section. In addition, our reconstructions are in good agreement with semi-quantitative paleo-water depth estimates from contemporaneous sediment records of the island of Rhodes. The reconstructed water depth changes reflect the presence of substantial long-term regional tectonic movements of Rhodes during the early Pleistocene.

## Supporting information

S1 File Taxonomic list of species used in this study. (XLSX)

S2 File Reaction norm code (R). (R)

S3 File Additional figures and tables. Evaluation of the reaction norm test, ordination analyses, abundances of species in the samples, ecological preferences of the species, transfer function evaluation.
(DOCX)
