# Supplementary material for: Testing the applicability of a benthic foraminiferal-based transfer function for the reconstruction of paleowater depth changes in Rhodes (Greece) during the early Pleistocene
Source: PLoS One. 2017 Nov 22;12(11):e0188447. doi: 10.1371/journal.pone.0188447 (PMC5699812; doi:10.1371/journal.pone.0188447)

Table A. **Evaluation of the reaction norm test.** Evaluation of the four reaction norm test methods by using simulated coenoclines representing truncated or full unimodal as well as linear responses. Given are the percentages of correct classifications for each test (GAM: generalized additive model).

| Test | linear | Truncated unimodal | Full unimodal |
| --- | --- | --- | --- |
| Goodness-of-fit | 96.5 | 88.5 | 100.0 |
| GAM | 95.5 | 87.0 | 100.0 |
| Binning | 9.5 | 100.0 | 100.0 |
| Piecewise regression | 99.0 | 21.5 | 100.0 |

Table B. **Statistical output.** Statistical output of the detrended correspondence analyses (DCA), canonical correspondence analyses (CCA), and goodness of fit statistics.

| **DCA-Combined modern data (data set used for TF)** | | |  |  |  |
| --- | --- | --- | --- | --- | --- |
| Axes | DCA1 | DCA2 | DCA3 | DCA4 |  |
| Eigenvalues | 0.663 | 0.629 | 0.274 | 0.142 |  |
| Axis lengths | 4.573 | 4.726 | 2.549 | 1.946 |  |
| **CCA-Combined modern data (data set used for TF)** | | |  |  |  |
| Axes | CCA1 | CA1 | CA2 | CA3 |  |
| Eigenvalue | 0.453 | 0.663 | 0.420 | 0.305 |  |
| Proportion Explained | 0.112 | 0.165 | 0.104 | 0.076 |  |
| Cumulative Proportion | 0.112 | 0.277 | 0.381 | 0.457 |  |
| **DCA-modern data Adriatic Sea (full data set)** | |  |  |  |  |
| Axes | DCA1 | DCA2 | DCA3 | DCA4 |  |
| Eigenvalues | 0.647 | 0.304 | 0.110 | 0.187 |  |
| Axis lengths | 5.020 | 3.565 | 1.648 | 1.914 |  |
| **CCA-modern data Adriatic Sea (full data set)** | |  |  |  |  |
| Axes | CCA1 | CCA2 | CCA3 | CA1 |  |
| Eigenvalue | 0.492 | 0.208 | 0.026 | 0.449 |  |
| Proportion Explained | 0.175 | 0.074 | 0.010 | 0.159 |  |
| Cumulative Proportion | 0.175 | 0.248 | 0.257 | 0.417 |  |
| **DCA-modern data Western Mediterranean Sea (full data set)** | | | |  |  |
| Axes | DCA1 | DCA2 | DCA3 | DCA4 |  |
| Eigenvalues | 0.459 | 0.314 | 0.120 | 0.093 |  |
| Axis lengths | 2.952 | 2.754 | 1.891 | 1.675 |  |
| **CCA-modern data Western Mediterranean Sea (full data set)** | | | |  |  |
| Axes | CCA1 | CCA2 | CCA3 | CA1 |  |
| Eigenvalue | 0.360 | 0.111 | 0.038 | 0.185 |  |
| Proportion Explained | 0.242 | 0.074 | 0.026 | 0.124 |  |
| Cumulative Proportion | 0.242 | 0.316 | 0.342 | 0.466 |  |
| **DCA-Pefka E fossil data (data set used for TF)** | |  |  |  |  |
| Axes | DCA1 | DCA2 | DCA3 | DCA4 |  |
| Eigenvalues | 0.148 | 0.109 | 0.083 | 0.070 |  |
| Axis lengths | 2.150 | 1.640 | 1.423 | 1.328 |  |
| **Goodness of fit-Combined modern and fossil data** | |  |  |  |  |
| CCA | 50% | 75% | 90% | 95% | 99% |
| Training Set | 2.5 | 4.5 | 6.3 | 8.6 | 20.4 |
| Passive - Pefka E profile | 10.0 | 12.0 | 13.9 | 15.6 | 23.6 |

Table C**. Summary of the reaction-norm tests applied to the modern dataset.** The table summarizes which proportion of species showed non-linear behavior according to each test statistic, and lists important species (with an abundance of ≥ 20% in at least three samples in the combined modern data set) with linear and non-linear coenoclines, respectively.

| Test | % non-linear | Non-linear species | Linear species |
| --- | --- | --- | --- |
| Goodness-of-fit | 57.1 | *Ammonia* spp. | *B. marginata* s.l. |
|  |  | *A. mamilla* | *C. pseudoungerianus* |
|  |  | *C. carinata* s.l. | *V. complanata* |
|  |  | *T. agglutinans* s.l. |  |
|  |  | *N. turgida* |  |
|  |  | *U. mediterranea* |  |
|  |  | *U. peregrina* |  |
| Piecewise regression | 41.4 (3 bins) | *Ammonia* spp. | *A. mamilla* |
|  |  | *C. carinata* s.l. | *B. marginata* s.l. |
|  |  | *N. turgida* | *C. pseudoungerianus* |
|  |  | *T. agglutinans* s.l. |  |
|  |  | *U. mediterranea* |  |
|  |  | *U. peregrina* |  |
|  |  | *V. complanata* |  |
| Binning | 85.7 (3 bins) | *Ammonia* spp. |  |
|  |  | *A. mamilla* |  |
|  |  | *B. marginata* s.l. |  |
|  |  | *C. carinata* s.l. |  |
|  |  | *C. pseudoungerianus* |  |
|  |  | *T. agglutinans* s.l. |  |
|  |  | *N. turgida* |  |
|  |  | *U. mediterranea* |  |
|  |  | *U. peregrina* |  |
|  |  | *V. complanata* |  |
| GAM | 71.4 | *Ammonia* spp. | *A. mamilla* |
|  |  | *C. carinata* s.l. | *B. marginata* s.l. |
|  |  | *C. pseudoungerianus* | *V. complanata* |
|  |  | *N. turgida* |  |
|  |  | *T. agglutinans* s.l. |  |
|  |  | *U. mediterranea* |  |
|  |  | *U. peregrina* |  |

Figure D. **CCA of the Adriatic Sea data set.** Canonical correspondence analysis (CCA) of the modern data set from the Adriatic Sea. Given in parentheses are the eigenvalues for the first two CCA axes (depth: water depth in m; sand: sand content in %; OC: organic carbon content in %).


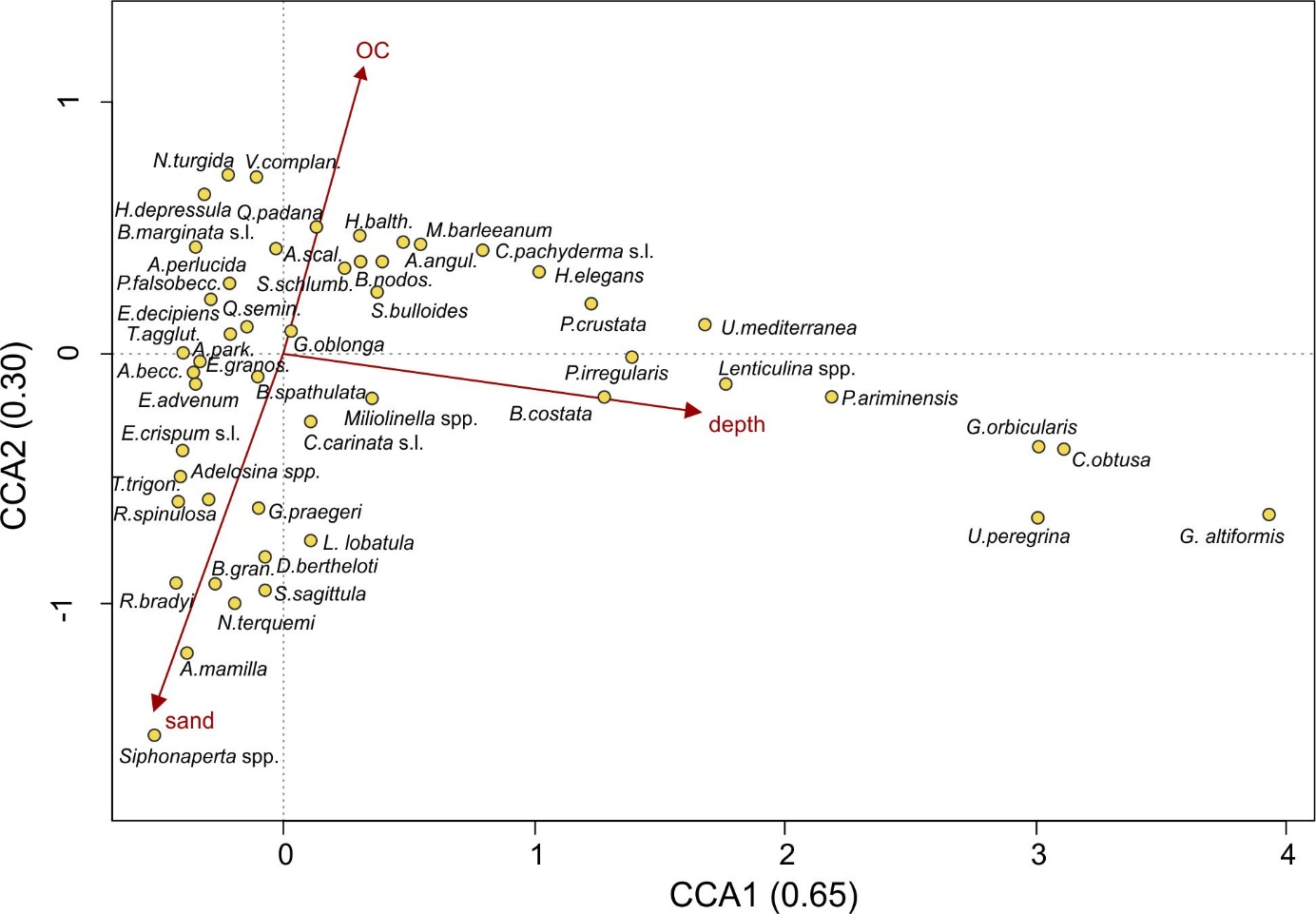


Figure E. **CCA of the Western Mediterranean Sea data set.** Canonical correspondence analysis (CCA) of the re-counted modern data set from the Western Mediterranean Sea. Given in parentheses are the eigenvalues for the first two CCA axes (depth: water depth in m; chl. *a*: chlorophyll a content (mg/m^3^); <63µm: fraction <63µm in per cent).


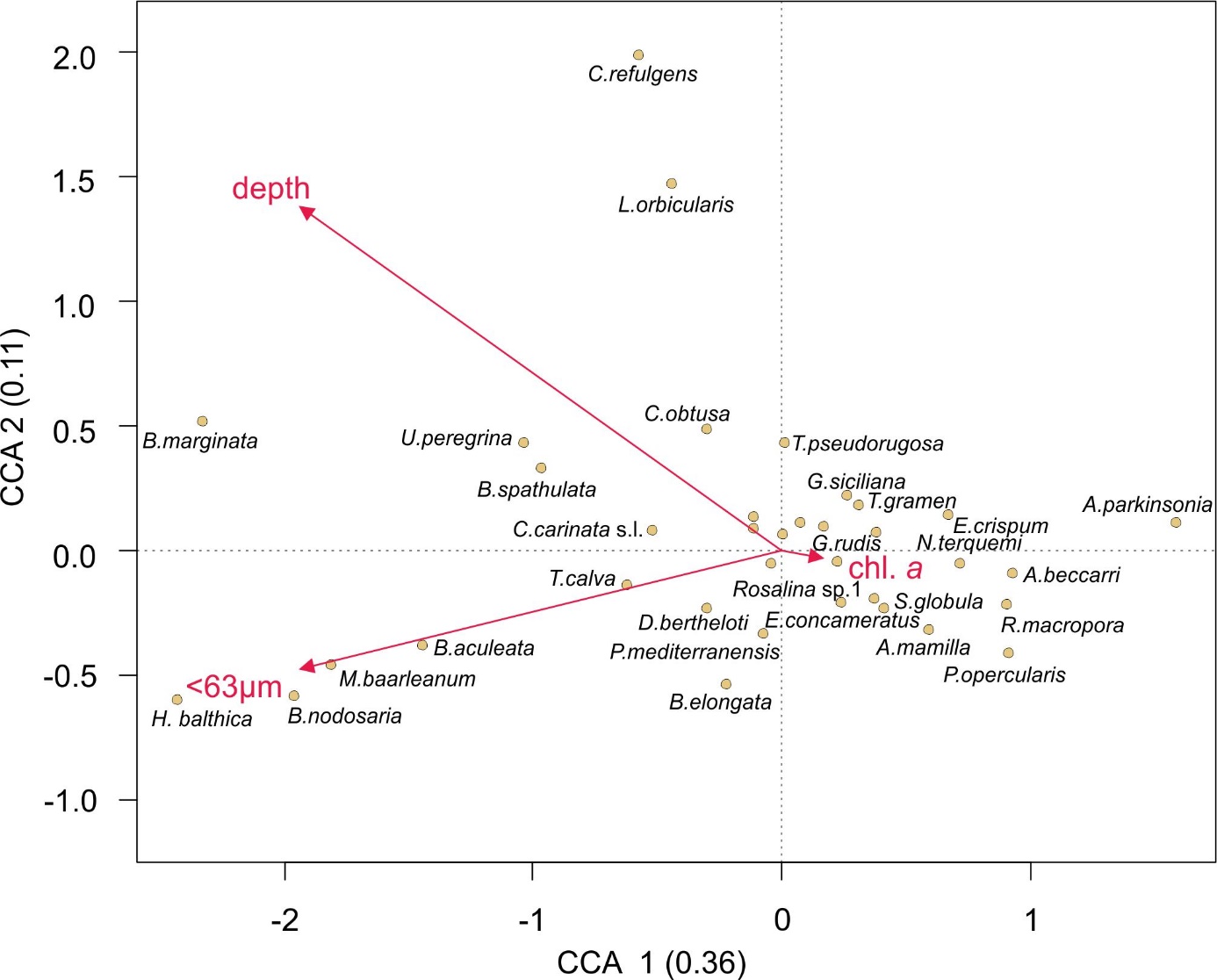


Figure F. **Infaunal fossil species.** Relative abundance of total, shallow, and intermediate to deep infaunal species in the Pefka E section versus age.


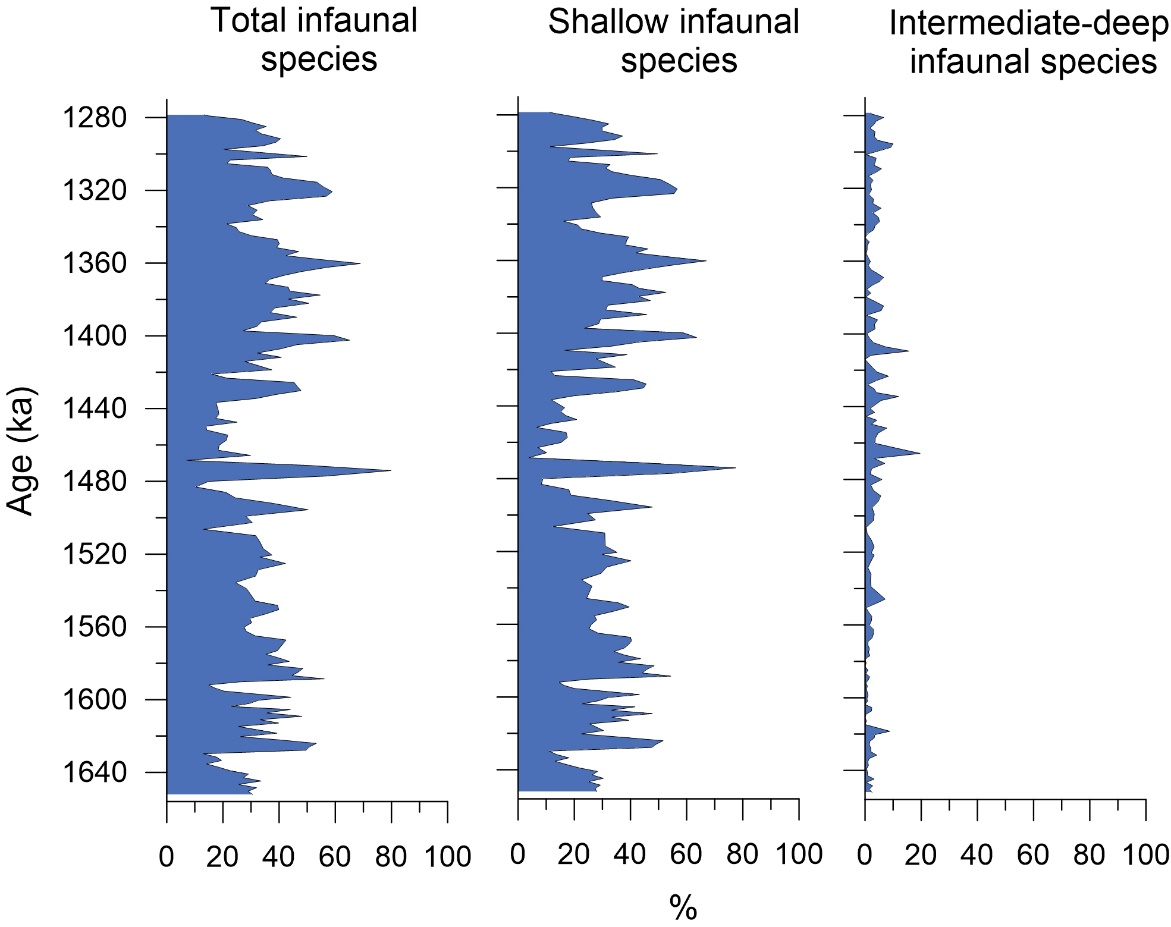


Figure G. **Optimum and tolerance of the modern species.** Optimum (points) and tolerance (error bars) of the modern species with respect to water depth. Calculations are based on Weighted Averaging. In bold are given species with a low effective occurrence (N2≤5) in the modern data set, indicating poorly defined optima.


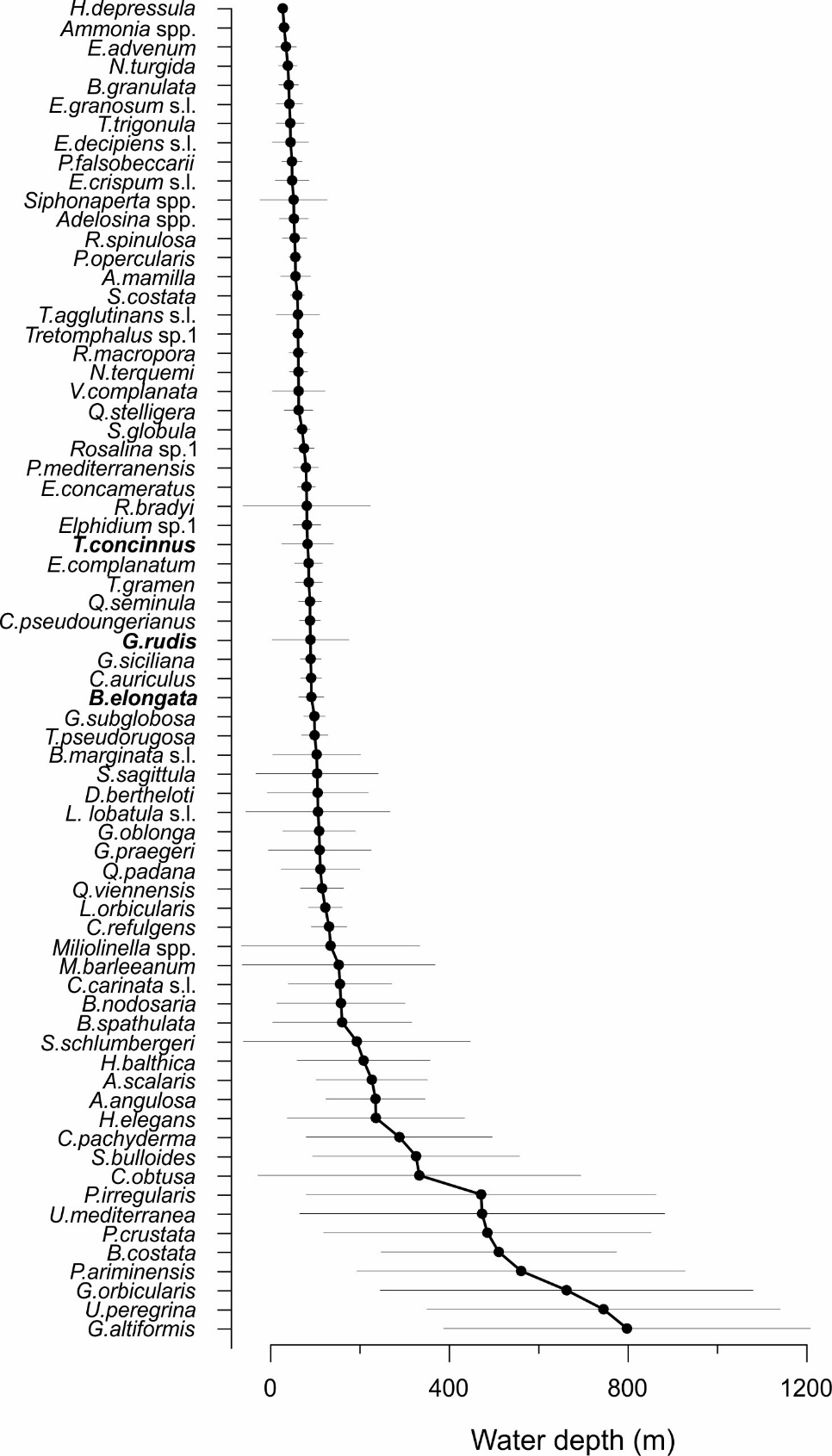


Fig H. **Randomization test.** Results of the randomization transfer function (TF) test applied to the modern data set. A: Measured versus reconstructed water depth, B: root mean squared error of prediction (RMSEP) and C: coefficients of determination for the 100 test TFs.


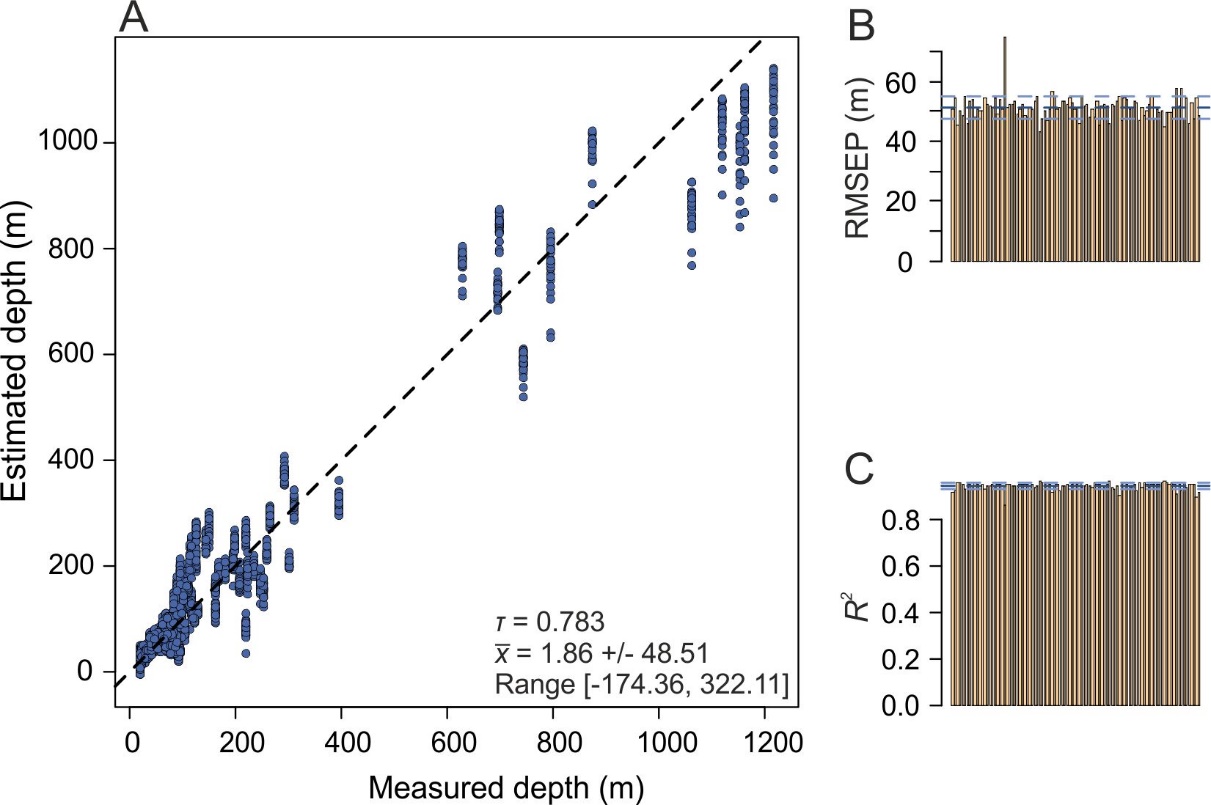


Figure I. **Coverage plot.** Maximum species abundance of the modern data set used for the transfer function versus their maximum abundance in the fossil Pefka E data set.


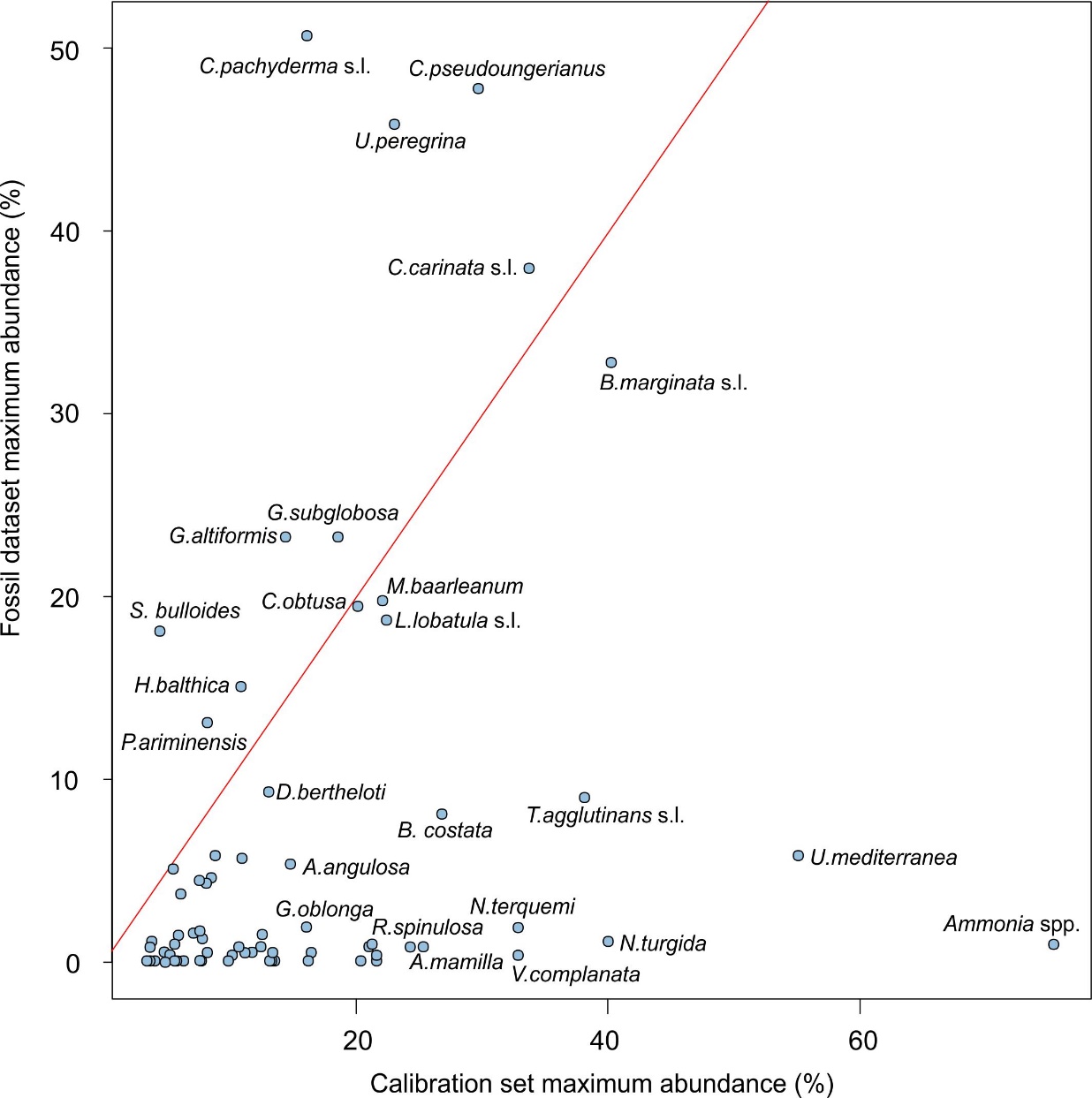


Figure J. **Jackknifing TF.** Root-mean-squared error of test transfer functions by excluding individual species by jackknifing applied to the Pefka E section.


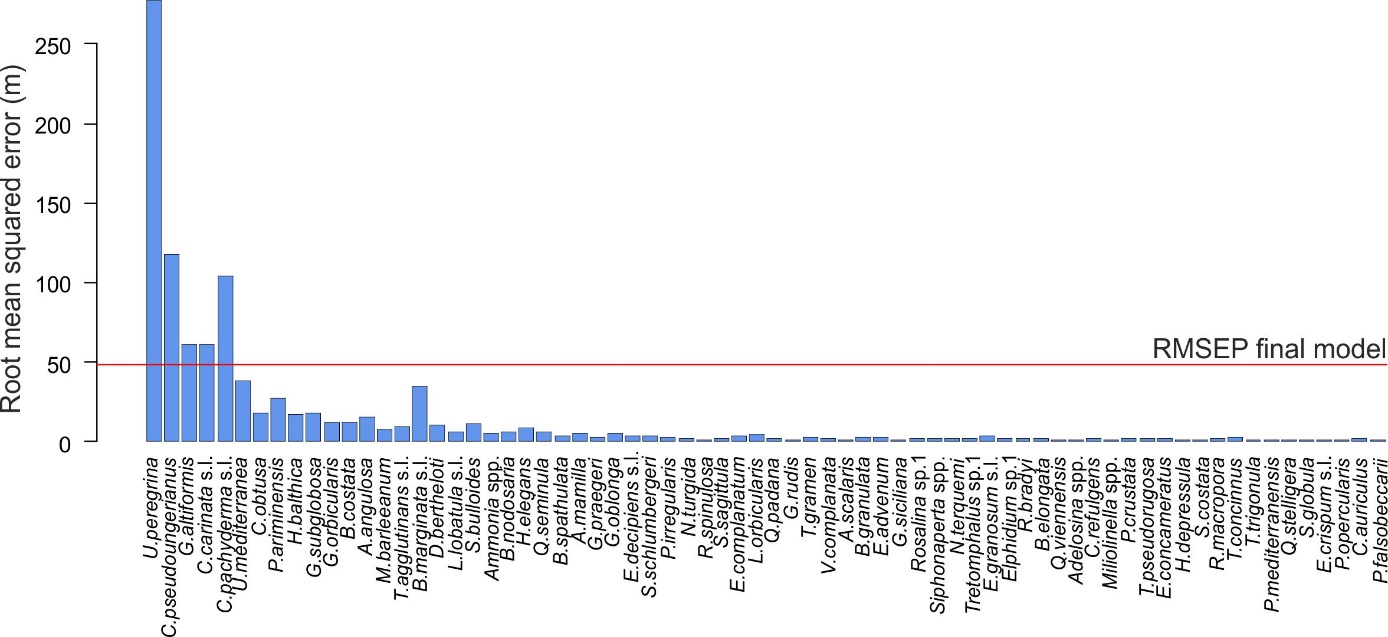


Figure K. **WA-PLS versus MAT.** Comparison of paleo-water depth estimates for the Pefka E section by using weighted averaging-partial least squares (WA-PLS) to those using the modern analogue technique (MAT).


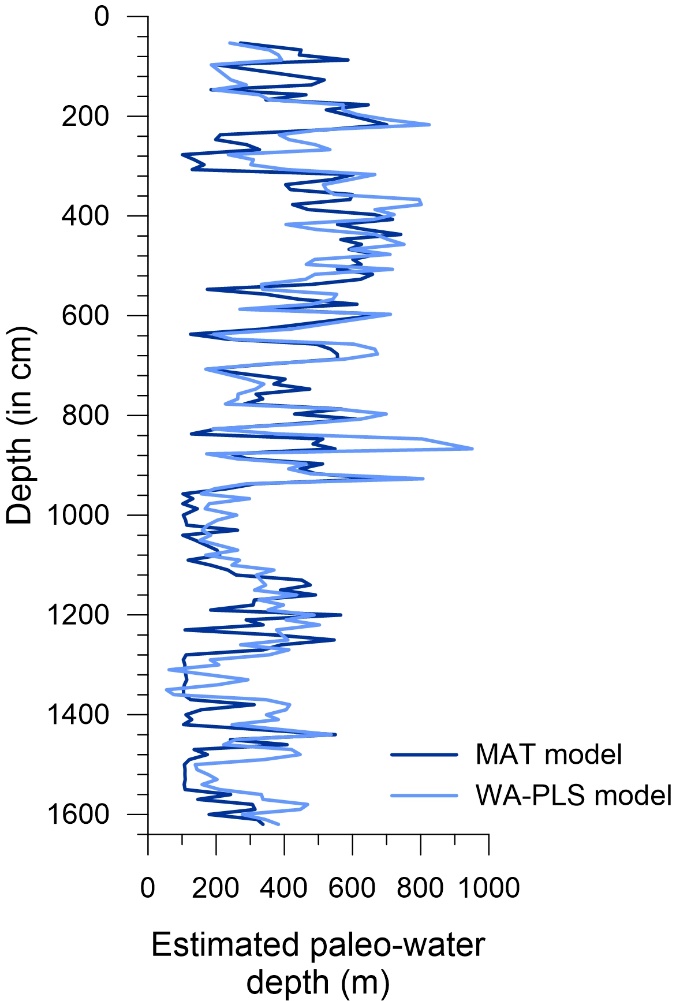

Supplement: S3 File — Evaluation of the reaction norm test, ordination analyses, abundances of species in the samples, ecological preferences of the species, transfer function evaluation. (DOCX) [file pone.0188447.s003.docx]
